# Comparative efficacy of aerobic training, resistance training, combined training, and multicomponent exercise on interleukin-6, C-reactive protein, and tumor necrosis factor-α levels in older adults (≥60 years): a systematic review and network meta-analysis

**DOI:** 10.3389/fphys.2026.1837579

**Published:** 2026-10-14

**Authors:** Wenchao Li, Rui Chu, Yeshou Xie, Yinuo Du, Tao Ni

**Affiliations:** 1 Capital University of Physical Education and Sports, Beijing, China; 2 Anhui Polytechnic University, Wuhu, Anhui, China; 3 Changzhou University, Changzhou, Jiangsu, China

**Keywords:** aged, exercise training, inflammatory biomarkers, interleukin-6, network meta-analysis

## Abstract

**Objective:**

To compare and rank the efficacy of four exercise training modalities—aerobic training (AT), resistance training (RT), combined training (AT+RT), and multicomponent exercise (MCEx)—on serum levels of interleukin-6 (IL-6), C-reactive protein (CRP), and tumor necrosis factor-α (TNF-α) in older adults, and to examine whether the optimal modality differs by participants’ health status (healthy vs. chronic disease).

**Methods:**

Strictly following the PRISMA guidelines, two researchers independently performed literature screening, methodological quality assessment, and data extraction in accordance with pre-established inclusion and exclusion criteria. A three-level meta-analysis was used to address the correlation of effect sizes in multi-arm trials; a frequentist random-effects network meta-analysis was conducted to compare and rank the intervention effects of aerobic training (AT), resistance training (RT), combined training (AT+RT), and multicomponent training (MCEx). The Cochrane Risk of Bias tool was used to assess the risk of bias of the included studies, and the GRADE framework was used to evaluate the certainty of evidence. The main effect size was the standardized mean difference (SMD) and its 95% confidence interval (95%CI), and the total I² statistic (I²_total) was used to quantify heterogeneity. In addition, double subgroup analysis was carried out based on different exercise intervention methods and different physical states of the elderly.

**Results:**

A total of 82 studies were included, involving 6493 participants, of which 3125 were in the control group and 3368 were in the experimental group, with a mean age of 71.04 years. Multilevel meta-analysis showed that exercise intervention could significantly reduce the levels of IL-6 (SMD = -0.31), CRP (SMD = -0.44), and TNF-α (SMD = -0.40) in the elderly. Subgroup analysis showed that AT+RT had the best improvement effect on IL-6, RT on CRP, and AT on TNF-α. Double subgroup analysis suggested that for the elderly with chronic diseases, RT and AT+RT could effectively improve their IL-6 and CRP levels; for healthy elderly, AT was an effective method to reduce their TNF-α levels. Network meta-analysis showed that AT ranked the best in reducing IL-6 (SUCRA = 84.6%) and TNF-α (SUCRA = 97.5%), while RT ranked the best in reducing CRP (SUCRA = 89.1%). Most of the included studies had unclear or high risk of bias, and the certainty of evidence for all three outcome indicators was very low based on GRADE assessment.

**Conclusion:**

Based on very low GRADE evidence, exercise interventions may reduce IL-6, CRP, and TNF-α in older adults, with aerobic training showing the highest probability of benefit for IL-6 and TNF-α, and resistance training for CRP. However, due to high risk of bias, heterogeneity, and publication bias, these findings are exploratory and require confirmation in high-quality trials.

**Systematic review registration:**

https://www.crd.york.ac.uk/PROSPERO/view/CRD420261320198, identifier CRD420261320198.

## 1 Introduction

With the acceleration of the global population aging process, in-depth exploration of the essential mechanisms of aging and search for effective approaches to delay aging and maintain the health of the elderly have become an important scientific research task. Among them, the concept of “inflammaging” was first proposed by Franceschi et al. to describe a chronic, low-grade, systemic inflammatory state that occurs with advancing age in the absence of overt infection or specific diseases (Furman et al., 2019).This state is characterized by a two- to four-fold elevation of circulating pro-inflammatory cytokines, including interleukin-6 (IL-6), tumor necrosis factor-alpha (TNF-α), and C-reactive protein (CRP), compared to young healthy adults (Karpuzoglu et al., 2025). This elevation is not caused by obvious acute infection or specific diseases, and its mechanism involves multiple factors including cellular senescence, senescence-associated secretory phenotype (SASP), mitochondrial dysfunction, gut microbiota dysbiosis, impaired clearance of cellular debris, as well as environment and lifestyle (Franceschi et al., 2000; Franceschi and Campisi, 2014; Zhu et al., 2021).A large number of studies have confirmed that the physiological changes induced by inflammaging, such as protein metabolism imbalance, hematopoietic function inhibition, neuroendocrine disorders, and endothelial dysfunction, make the chronic low-grade inflammatory state in the elderly closely associated with the occurrence and development of various age-related diseases, such as cardiovascular diseases, type 2 diabetes, Alzheimer’s disease, Parkinson’s disease, osteoporosis, sarcopenia, frailty syndrome, and various malignant tumors (Bourdel-Marchasson et al., 2010; Mitchell et al., 2012; Bie and Tao, 2025).Therefore, effectively reducing the expression levels of core pro-inflammatory factors such as serum IL-6, CRP, and TNF-α in the elderly and improving the state of inflammaging have become key entry points to delay the aging process, reduce the risk of age-related diseases, and improve the quality of life of the elderly population.In this study, older adults are defined as individuals with a mean age of 60 years or older, consistent with the inclusion criteria of the systematic review.

Various pharmacological approaches have been explored for managing inflammaging in the elderly, including non-steroidal anti-inflammatory drugs, statins, and interleukin inhibitors. While these interventions show some efficacy, they are associated with notable limitations including various side effects and risks of polypharmacy (Corsonello et al., 2010; Woods et al., 2012). For nutritional intervention, such as intake of a large amount of fruits and vegetables, whole grains, beans, nuts, olive oil, etc., while generally safe, its anti-inflammatory effects are modest and long-term adherence remains challenging (Cederholm, 2015; Fulop et al., 2018). In addition, as a non-pharmacological intervention method, exercise intervention has shown good effects in improving hormone levels and physical activity ability of the elderly, and has the advantages of high safety and few side effects (Landi et al., 2014). Despite the accumulating evidence, several critical gaps remain unresolved. First, existing meta-analyses have yielded conflicting conclusions regarding the anti-inflammatory effect of exercise in older adults—some reporting significant reductions in IL-6, CRP, and TNF-α (Khalafi et al., 2023; Sheldon et al., 2026), while others found no significant effects (Sardeli et al., 2018; Nejatian Hoseinpour et al., 2025). This discrepancy may stem from differences in study populations, exercise protocols, and analytical approaches. Second, most prior meta-analyses focused on a single exercise modality (e.g., resistance training alone) or pooled all exercise types together, thereby failing to provide comparative efficacy rankings across different modalities (Zhao et al., 2022; Cheng and Yang, 2024). Third, no previous meta-analysis has systematically examined whether the optimal exercise modality differs according to participants’ health status (healthy vs. chronic disease), despite the plausible biological premise that chronic inflammatory conditions may respond differently to various exercise stimuli. Fourth, prior meta-analyses rarely accounted for the statistical dependency arising from multi-arm trials (e.g., studies comparing AT, RT, and CON simultaneously), which can artificially inflate precision and bias effect estimates.

To address these gaps, the present study integrates existing RCTs using a combination of three advanced methodological approaches: (1) a three-level multilevel meta-analysis that properly handles effect size dependency from multi-arm trials; (2) a frequentist network meta-analysis to compare and rank the relative efficacy of four exercise modalities (AT, RT, AT+RT, and MCEx) on IL-6, CRP, and TNF-α levels; and (3) a double subgroup analysis stratified by both exercise modality and participants’ health status (healthy vs. chronic disease) to determine whether treatment effects are modified by underlying inflammatory burden.

The primary objectives are: (a)to evaluate the overall anti-inflammatory effect of AT, RT, AT+RT, and MCEx on IL-6, CRP, and TNF-α levels in older adults (defined as mean age ≥60 years); (b) to rank the four exercise modalities to identify the most effective intervention for each inflammatory marker; and (c) to determine whether the optimal modality differs by health status (healthy vs. chronic disease) through double subgroup analysise. The findings are expected to offer a robust, evidence-based reference for non-pharmacological management of inflammaging in the elderly, addressing the limitations of prior work through improved statistical rigor and clinical granularity.

## 2 Methods

This study was conducted in accordance with the PRISMA (Preferred Reporting Items for Systematic Reviews and Meta-Analyses) statement guidelines (Liberati et al., 2009). The study protocol has been registered with the PROSPERO database (Centre for Reviews and Dissemination, University of York) under the registration number CRD420261320198, available at: https://www.crd.york.ac.uk/PROSPERO/view/CRD420261320198.

### 2.1 Search strategy

Literature retrieval was independently performed by two investigators (YND and TN) in a double-blind manner. The search time range was set from the inception of each database to January 30, 2026. Both English and Chinese databases were searched. English databases included Web of Science, Cochrane Library, PubMed, EMBASE, and Scopus, while Chinese databases mainly comprised China National Knowledge Infrastructure (CNKI) and Wanfang Data Knowledge Service Platform. A combined search strategy using subject headings and free words was adopted. The core search concepts were divided into three categories: ① related inflammatory factors, such as IL-6, IL-10, CRP, and TNF-α; ② related exercise interventions, such as resistance training, aerobic training, and multicomponent training; ③ target study population. The detailed search equations and procedures are shown in **Tables 1**, **2**.

**Table 1:** List of Specific search terms.

Concept groups	Search terms
Inflammatory Biomarker	Pro-Inflammatory, Interleukin-6, tumor necrosis factor alpha, C reactive protein
Exercise Intervention	Exercise, Physical Exercise, Aerobic Exercise, Resistance Training, Endurance Training, Exercise Training, Multi component training, Combined training
Target population	senior citizen, Aged, Elderly
Research type	Randomized controlled trial

**Table 2:** List of search syntax.

Search strategy	Search terms
#1	Exercise/OR Physical Exercise/OR Aerobic Exercise/OR Multi component training/OR Exercise Training/OR Resistance Training/OR Endurance Training/OR Combined training
#2	Pro-Inflammatory/OR Interleukin-6/OR tumor necrosis factor alpha/OR C reactive protein
#3	senior citizen/OR Aged/OR Elderly/
#4	Randomized controlled trial
#5	#1 AND #2 AND #3 AND #4

After literature collection was completed, duplicate records were first removed using bibliographic management software such as EndNote. Subsequently, preliminary screening of titles and abstracts was performed in accordance with the pre-established inclusion and exclusion criteria, and obviously irrelevant studies were excluded. Full texts were obtained for articles that passed the preliminary screening, and two investigators (YND and TN) independently conducted the second screening, methodological quality assessment and data extraction. Any discrepancies between the two investigators were resolved through discussion organized by the corresponding author until a consensus was reached.

Inter-rater reliability. To assess the consistency of the screening and data extraction processes, Cohen’s kappa (κ) coefficient was calculated for each stage. For title/abstract screening, a pilot test of 200 randomly selected records was conducted, with κ ≥ 0.80 considered as “almost perfect” agreement. For full-text screening and data extraction, κ was calculated based on independent assessments of all included studies and a random sample of excluded studies (10% of excluded full texts). Interpretation followed Landis and Koch (1977): κ < 0.00 = poor, 0.00–0.20 = slight, 0.21–0.40 = fair, 0.41–0.60 = moderate, 0.61–0.80 = substantial, 0.81–1.00 = almost perfect. Discrepancies were resolved by discussion; where disagreement persisted, a third reviewer (WL) made the final decision.

### 2.2 Inclusion and exclusion criteria

According to the Cochrane PICOS principle, the inclusion and exclusion criteria for literature established in this study are as follows:

Inclusion criteria:

1. Study Population: Community-dwelling, nursing home-residing, or outpatient elderly individuals with a mean age of ≥ 60 years. If a study included a broader age range, data on the subgroup aged ≥ 60 years must be extractable independently. Eligible participants included healthy elderly individuals, frail elderly individuals, and those with stable chronic diseases (e.g., hypertension, type 2 diabetes mellitus, osteoarthritis) but without acute infection.
2. Intervention and Control Measures: The experimental group received structured exercise training with a clear protocol and a duration of ≥ 4 weeks, including but not limited to: aerobic training, resistance training, high-intensity interval training (HIIT), balance training, flexibility training, Tai Chi, yoga, and multicomponent training combining the abovementioned exercises. The control group was either a non-exercise intervention group (e.g., health education, routine care, waiting list) or a group receiving another type/intensity of exercise intervention.
3. Outcome Measures: Studies must report at least one of the following major inflammatory markers with baseline and post-intervention data (or changes before and after intervention): high-sensitivity C-reactive protein (hs-CRP) or C-reactive protein (CRP), interleukin-6 (IL-6), tumor necrosis factor-α (TNF-α).
4. Study Type: Only randomized controlled trials (RCTs) were included.
5. Publication Type and Language: Full-text articles published in peer-reviewed journals in Chinese or English were included; conference abstracts, reviews, letters, case reports, and other non-full-text literatures were excluded.

Exclusion criteria:

1. Exclusion Criteria for Study Population and Related Studies: Studies with a mean age of study population < 60 years; acute inpatients, and patients with active infection, autoimmune diseases, advanced cancer, or other diseases known to cause severe systemic inflammation; studies focusing on acute exercise response (single bout of exercise).
2. Exclusion Criteria for Interventions: Studies mainly based on non-exercise interventions (e.g., only nutritional supplementation, drug intervention, etc.); combined intervention studies that cannot distinguish the effect of exercise (e.g., exercise combined with dietary intervention without a separate exercise control group); studies that only provide “physical activity advice” without clearly describing specific training protocols.
3. Exclusion Criteria for Study Design: Single-group pre-test and post-test studies without a control group.
4. Exclusion Criteria for Outcome Measures: Studies that only report other secondary inflammatory markers (e.g., IL-1β, IL-10, etc.) or do not report extractable quantitative data.
5. Exclusion Criteria for Study Type and Publication Form: Non-randomized studies, observational studies, case reports, reviews, systematic reviews/meta-analyses, study protocols, conference abstracts, and articles with inaccessible full text or relevant data; gray literature, dissertations, book chapters (unless they contain original randomized controlled trial data).

### 2.3 Exercise categories

According to the characteristics of the exercise intervention protocols in the included RCTs, this study classified them into four categories: aerobic training (AT), resistance training (RT), combined training (AT+RT), and multicomponent training (MCEx). The specific definitions of each exercise type are shown in **Table 3**.

**Table 3:** The classifications of exercise training.

Type	Definition	
CON	No exercise or slight stretching	
Aerobic training (AT)	Type: Any continuous traditional mode of aerobic training only (e.g., walking, running, cycling, rowing, aerobics, and elliptical exercise) (O'Donoghue et al., 2021).	Intensity: 45%–65%VO2max or >50%–65% HRR or 65%–75% MHR
RT	Intensity: ≥50% 1RM;Type: Any mode of resistance training, including circuit- based programs (e.g., free weights, weights machines, and resistance bands) (O'Donoghue et al., 2021).	
AT + RT	Aerobic training combined with resistance training only (no additional components such as balance or flexibility training)	
Multicomponent Exercise (MCEx)	A structured exercise regimen that systematically integrates three or more distinct exercise types—typically including aerobic, resistance, and at least one of balance or flexibility training. Interventions consisting of only aerobic plus resistance training (without a third component) were classified as AT+RT, not MCEx. This definition ensures mutual exclusivity with AT+RT (Ordoñez-Mora et al., 2025).	

Mixed or unclear protocols. If an intervention could not be unambiguously assigned to one of the above categories due to insufficient description or conflicting information, the study authors were contacted for clarification. If no clarification could be obtained (within two email attempts), the study was excluded from the network meta-analysis for the relevant comparison. In the present review, all 82 included studies provided sufficient detail to allow unambiguous classification according to the above rules; no study was excluded due to protocol ambiguity.

### 2.4 Data extraction

After completing the literature screening, two investigators (Du Yinuo and Ni Tao) independently extracted data from the included studies in a double-blind manner. The extracted data mainly covered the following dimensions:

1. Basic information of the literature: first author’s name, year of publication, etc.;
2. Subject information: sample size, mean age, physical status (healthy, with chronic diseases, etc.), etc.;
3. Intervention information: specific exercise training protocols, including core intervention parameters such as exercise type, exercise mode, training tools, intervention duration, training frequency, single training session duration, and intensity;
4. Study results: for the primary outcome measures, the following data were extracted from each intervention group and control group: baseline mean and standard deviation (SD), post-intervention mean and SD, and number of participants. If the study report directly provided the mean change (post-intervention minus baseline) and its SD, these data were extracted directly; if not, they were calculated based on the baseline and post-intervention statistical data. To calculate the SD of the change, this study assumed a correlation coefficient (r) of 0.5 between pre-intervention and post-intervention measurements within each group, in accordance with the recommendations of the Cochrane Handbook for Systematic Reviews (Deeks et al., 2023). The SD of the change was calculated using the following formula: SD_change = (SD_pre² + SD_post² - 2 × r × SD_pre × SD_post), where r = 0.5.During the data extraction process, any discrepancies between the two investigators were resolved through thematic meetings organized by the corresponding author to reach a final consensus.

### 2.5 Risk of bias and GRADE assessment

This study used the original Cochrane Risk of Bias Tool (The Cochrane Collaboration’s tool for assessing risk of bias in randomised trials) (Higgins et al., 2011) to assess the risk of bias in included studies, with independent assessment conducted by two researchers (YND and TN). Any discrepancies in the assessment results were resolved through discussions organized by the corresponding author. The assessment covered the following six core domains:

1. Selection bias during randomization: Assessing whether the allocation of participants was truly random and whether allocation concealment was adequate (i.e., whether the allocation sequence was effectively hidden before group assignment), to avoid selection bias caused by researchers or participants predicting group assignments.
2. Performance bias due to deviations from intended interventions: Assessing whether blinding was successfully implemented for participants and intervention providers (e.g., medical staff); if no blinding was applied, determining whether there were systematic deviations from the intended intervention that could affect results.
3. Attrition bias due to missing outcome data: Judging the completeness of outcome data; if data were incomplete, analyzing whether the proportion and reasons for missing data were related to true outcomes, which might lead to bias in estimating between-group effect sizes.
4. Detection bias due to inappropriate outcome measurement: Assessing whether the measurement methods for outcome indicators were objective and reliable, and whether outcome assessors were blinded to participants’ group information, to avoid subjective judgments being influenced by group information.
5. Reporting bias due to selective reporting of results: Judging whether the results presented in the final report were based on pre-specified study protocols and analysis plans, rather than results derived from “data dredging” or “selective reporting.”
6. Other potential sources of bias: This domain is not an independent source of bias but a logical integration of the assessment results of the first 5 core domains.

Overall risk of bias judgment.Following the Cochrane Handbook’s guidance, we did not use a numerical scoring system or calculate a summary score. Instead, we applied the “worst domain” principle (also known as the “shortest plank” principle) to determine the overall risk of bias for each study. Specifically:

If any of the six domains was rated as “high risk”, the overall risk of bias was judged as “high risk”.

If no domain was “high risk” but at least one domain was rated as “some concerns” (or “unclear” for RoB 1), the overall risk was judged as “some concerns”.

Only when all domains were rated as “low risk” was the overall risk of bias judged as “low risk”.

This approach aligns with Cochrane’s recommended method for synthesizing domain-level judgments without resorting to invalid scoring systems.

The certainty (quality) of evidence for the primary outcome was assessed using the Grading of Recommendations, Assessment, Development, and Evaluation (GRADE) framework. The certainty was rated as high, moderate, low, or very low based on the following five domains: risk of bias (assessed using the RoB tool), inconsistency (statistical heterogeneity), indirectness (of populations, interventions, and outcomes), imprecision (of the effect estimate, based on sample size and confidence interval width), and publication bias. The assessment was performed independently by two reviewers (YND and TN), with disagreements resolved through discussion or consultation with a third reviewer. A ‘Summary of Findings’ (SoF) table was generated to present the key findings along with the GRADE ratings and reasons for downgrading.

### 2.6 Statistical analysis

To appropriately address the dependency among effect sizes (where some studies were multi-arm trials), a multilevel meta-analysis (MLMA) was employed as the primary analytical framework. All statistical analyses were conducted using R software (version 4.4.1) and the metafor package (version 4.8.0), with a three-level random-effects model fitted: effect sizes (level 1) were nested within intervention arms (level 2), which were in turn nested within studies (level 3). By estimating the variance at the study level, this model structure explicitly accounts for the correlation between effect sizes derived from the same study (e.g., effect sizes generated from multi-group trials with a shared control group), thereby avoiding the artificial inflation of precision (narrowing of confidence intervals) that would occur if these correlated comparisons were treated as independent (Phillippo et al., 2025).

The primary effect size for the meta-analysis was the Standardized Mean Difference (SMD). Although all included studies measured relevant outcome indicators, the units of measurement used to report the results were inconsistent. The SMD expresses the between-group difference based on the pooled standard deviation (SD), which allows for the combination of outcome indicators measured on different scales into a single, unitless metric. This approach is standard and recommended when studies assess the same underlying construct but use different measurement units that are difficult to convert into a single common unit based on the reported data.

Effect sizes were expressed as SMD with 95% Confidence Intervals (95% CI). The Hartung-Knapp-Sidik-Jonkman (HKSJ) method was used to adjust the confidence intervals for studies with small sample sizes. Heterogeneity was quantified using the total I² statistic (I²_total), which reflects the proportion of the total variance attributable to variations at both levels.

In addition to the primary analysis, the following additional analyses were performed:

1. Subgroup Analysis: A random-effects model was used to compare the effects of different exercise types on the primary outcome indicators. Meanwhile, a double subgroup analysis was conducted to explore the intervention effects of different exercise intervention methods on elderly individuals with different physical statuses for different outcome indicators.
2. Publication Bias Assessment: Potential small-study effects or publication bias were evaluated using three complementary approaches. First, a rank correlation test (Kendall’s τ) adapted for multilevel models was used as the primary method, as it accounts for the dependency among effect sizes arising from multi-arm trials. Second, a traditional Egger’s regression test was performed for descriptive comparison, but we acknowledge that its assumption of independent effect sizes is violated in the presence of dependent effect sizes (e.g., multiple comparisons from the same study). Therefore, conclusions regarding publication bias are based primarily on the Kendall’s τ test and visual inspection of funnel plots, with Egger’s test results reported only as supplementary information. Third, funnel plots were visually inspected for asymmetry. Asymmetry was defined as visual imbalance and statistically supported by a significant Kendall’s τ (p < 0.10) when accompanied by clear visual asymmetry.
3. Sensitivity Analysis: The robustness of the pooled effect size was tested by varying the assumed within-study correlation coefficient (ρ = 0.3, 0.5, 0.7) between correlated effect sizes. A leave-one-out sensitivity analysis was also conducted by iteratively removing each study to assess its impact on the overall results. This method allows us to evaluate whether the SMD is sensitive to changes in the imputed correlation. In addition, the leave-one-out sensitivity analysis can also test the influence of individual studies on the overall effect size.

In the primary three-level model, dependency from shared-control multi-arm trials was accommodated structurally via hierarchical variance components (effect sizes nested within arms nested within studies), without imposing a fixed correlation on the sampling covariance matrix. The assumed correlations (ρ = 0.3, 0.5, 0.7) were used only in sensitivity analyses to construct the sampling covariance matrix for shared-control comparisons only; they were not applied to multiple outcomes, as each outcome was analyzed separately.

Potential sources of heterogeneity (exercise modality, health status) were pre-specified in the protocol. Subgroup differences were formally tested using appropriate statistical methods, and the p-values are reported in Section 3.4.

In addition to the multilevel meta-analysis, a network meta-analysis (NMA) was conducted using Stata software (version 15, StataCorp, College Station, TX, USA) to evaluate the comparative efficacy of different exercise modalities on inflammatory biomarker levels. A random-effects NMA within a frequentist framework was employed to synthesize direct and indirect evidence, while preserving the randomization of the included studies. A network plot was generated to illustrate the evidence structure, with node size reflecting the number of participants and edge thickness representing the number of studies. Inconsistency between direct and indirect evidence was assessed globally using the design-by-treatment interaction model and locally via the node-splitting method. The relative treatment effects were presented as standardized mean differences (SMDs) with 95% confidence intervals (95% CI). Treatment hierarchies were established using the surface under the cumulative ranking curve (SUCRA) and mean ranks. Furthermore, a comparison-adjusted funnel plot was used to assess the potential for small-study effects within the network.

To quantitatively assess transitivity, we compared the distribution of key potential effect modifiers across the different direct comparison types (e.g., AT vs. CON, RT vs. CON, AT+RT vs. CON, MCEx vs. CON, and head-to-head comparisons between active interventions). For continuous variables (mean age, baseline IL-6/CRP/TNF-α levels, intervention duration, exercise intensity), we performed one-way analysis of variance (ANOVA) or Kruskal-Wallis tests as appropriate. For categorical variables (proportion of participants with chronic diseases, risk of bias rating), chi-square tests were used. No statistically significant differences were found across comparison types for any of these variables (all p > 0.05), supporting the plausibility of the transitivity assumption. Detailed results are presented in **Supplementary Table S1**.

Network balance diagnostics. Network structure and coherence diagnostics. To characterize the evidence network, we calculated the following metrics for each outcome network: (i) the mean number of studies per comparison; (ii) the percentage of comparisons with at least one direct study (network density), reflecting network structure and balance; and (iii) the I² statistic for inconsistency, which quantifies the degree of heterogeneity within each direct comparison and was calculated as I² = max(0, (Q − df)/Q) × 100%, where Q is Cochran’s Q statistic for heterogeneity within each direct comparison and df is its degrees of freedom. Higher I² values indicate greater within-comparison heterogeneity. Importantly, network density (structural property) and I² for inconsistency (coherence property) represent distinct aspects of the evidence network and are reported as complementary diagnostics.For IL-6, the network had 10 possible comparisons, of which 7 (70%) were informed by direct evidence, with a mean of 3.4 studies per direct comparison. For CRP, 8 of 10 comparisons (80%) were directly informed, mean 2.9 studies per comparison. For TNF-α, only 6 of 10 comparisons (60%) were directly informed, with a mean of 2.2 studies per comparison, indicating a sparser network. These diagnostics are summarized in **Supplementary Table S2**. The relatively sparse network for TNF-α suggests that indirect comparisons for this outcome should be interpreted with greater caution.

The relationship between the two approaches is as follows: MLMA provides the direct effect estimates (including subgroup-specific estimates), while NMA synthesizes both direct and indirect evidence to produce a unified ranking. Both analyses used the same inclusion criteria, outcome definitions, and effect size metric (SMD). Results from the MLMA (e.g., direct comparisons) are reported first, followed by the NMA results (rankings and indirect comparisons). Discrepancies between the two approaches are noted and discussed.

## 3 Results

### 3.1 Studies search results

**Figure 1** shows the literature screening flow chart. A total of 3999 potentially relevant studies were initially retrieved, including 3988 from database searches and 11 from reference list tracing. After removing 129 duplicate studies using bibliographic management software, the remaining 3870 studies entered the screening process. A total of 3650 studies were excluded after preliminary screening of titles and abstracts; after further obtaining and reviewing the full texts, 138 additional studies were excluded, and finally 82 studies were included in the quantitative analysis.

**Figure 1:**
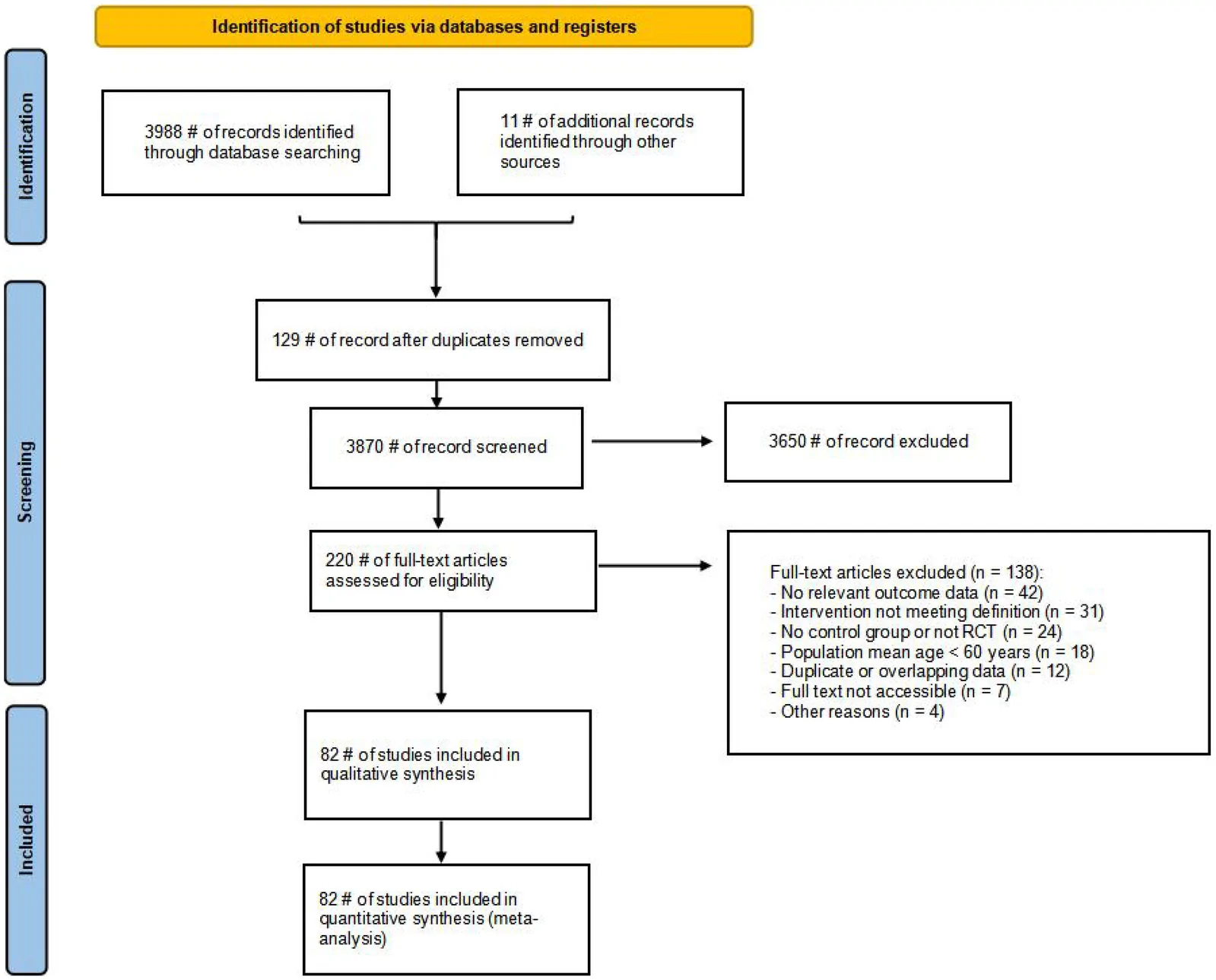
PRISMA flow diagram of the study selection process.

The inter-rater reliability for title/abstract screening was almost perfect (Cohen’s κ = 0.86, 95% CI: 0.82–0.90). For full-text screening, the agreement remained almost perfect (κ = 0.83, 95% CI: 0.77–0.89). For data extraction of key variables (e.g., sample size, mean age, intervention parameters, outcome values), Cohen’s κ ranged from 0.79 to 0.94 across different variables, indicating substantial to almost perfect agreement. All discrepancies were resolved through discussion, with consensus reached for all items.

### 3.2 Characteristics of the studies

A total of 82 studies were included. Because some studies were multi-arm trials with multiple intervention arms (e.g., comparing both AT and RT against a common control), these 82 studies contributed a total of 88 intervention-arm observations across the four exercise modalities. **Table 4** systematically summarizes the basic characteristics of the included studies, including author information, year of publication, characteristics of study subjects (mean age, sample size, health status), characteristics of intervention measures (control measures, intervention methods, intervention duration, frequency and intensity), and the involved outcome indicators. Specifically, among the 82 included studies, 40 studies focused on healthy elderly individuals, and 42 studies focused on elderly individuals with chronic diseases, among which the number of studies targeting elderly individuals with sarcopenia was the largest (8 studies); the mean age of the included subjects was 71.04 years old, with a total sample size of 6493 people, including 3125 in the control group and 3368 in the experimental group; regarding control measures, the number of studies using “no exercise” as the control was the largest (37 studies); regarding exercise intervention measures, 29 studies adopted AT, 34 studies adopted resistance training (RT), 13 studies adopted combined training (AT+RT), and 12 studies adopted MCEx; the overall intervention duration ranged from 4 to 72 weeks, among which the number of studies with 12-week intervention duration was the largest (29 studies); the intervention frequency ranged from 2 to 6 times per week, among which the intervention frequency of 3 times per week was the most widely used (53 studies); regarding outcome indicators, 67 studies analyzed IL-6, 58 studies analyzed CRP, and 48 studies analyzed TNF-α.

**Table 4:** Characteristics of included studies.

Study	Participant status	n	Age	Intervention characteristics					Outcome
				CG	EG	Intervention duration (weeks)	Intervention frequency (time/week)	Intensity	
Chen et al.(2022) (Chen and Yi, 2022)	Stable COPD	CG:12;EG1:12;EG2:13	CG:64.6 ± 3.8;EG1:66.1 ± 4.9;EG2:65.5 ± 3.3	Conventional Treatment	AT+RT	36	3	60%-85%HRmax	IL-6
Uzeloto et al.(2022) (Uzeloto et al., 2022)	Stable COPD	CG:8;EG:15	CG:72.25 ± 6.86;EG:68 ± 5.96	Conventional Treatment	AT+RT	8	2	AT: Borg4-6; RT:60%-80%1-RM	IL-6, TNF-α
Schlager et al.(2012) (Schlager et al., 2012)	Intermittent Claudication	CG:26;EG:27	CG:70.7 ± 10.6;EG:68.4 ± 7.5	Conventional Treatment	AT	24	2	–	CRP, IL-6
Haider et al.(2017) (Haider et al., 2017)	Frailty	CG:23;EG:35	CG:81.4 ± 8.7;EG:83.1 ± 7.8	No exercise	RT	12	2	18–20 repetitions/set	TNF, CRP
Alghadir et al.(2016) (Alghadir et al., 2016)	Healthy	CG:50;EG:50	CG:67.3 ± 2.8;EG:66.8 ± 3.7	No exercise	AT	24	3	60%-70%HRmax	CRP
Son etal.(2024) (Son et al., 2024)	Obesity	CG:12;EG:14	CG:71.17 ± 4.66;EG:71.67 ± 2.74	No exercise	AT	12	3	RPE11-14	TNF
Kemmler et al.(2009) (Kemmler et al., 2009)	Metabolic Syndrome	CG:32;EG:33	CG:69.5 ± 4.3;EG:68.7 ± 3.4	No exercise	AT+RT	48	4	–	CRP
Nishijima et al.(2007) (Nishijima et al., 2007)	Cardiovascular Disease	CG:280;EG:281	CG:66.9 ± 6.9;EG:67.0 ± 6.7	Health Education	AT	24	2—4	VO2max40-70%	CRP
Martins et al.(2010) (Martins et al., 2010b)	Healthy	CG:32;EG:32	CG:76 ± 8;EG:76 ± 8	No exercise	AT+RT	16	3	71-85% HRR	CRP
Messier et al.(2013) (Messier et al., 2013)	Obesity	CG:150;EG:150	CG:66 ± 6;EG:66 ± 6	Nutritional Intervention	AT+RT	72	3	Moderate Intensity	IL-6
Cunha et al.(2021) (Cunha et al., 2021)	Healthy	CG:18;EG1:19;EG2:18	CG:69.0 ± 4.2;EG1:70.3 ± 6.3;EG2:68.7 ± 4.7	No exercise	RT	12	3	LV:10-15RM;HV:3组8-12RM	CRP
Tan et al.(2023) (Tan et al., 2023)	Frailty	CG:187;EG:87	CG:71.69 ± 4.99;EG:73.39 ± 5.20	Health Education	MCEx	24	2	–	TNF, IL-6
Tomeleri et al.(2018) (Tomeleri et al., 2018b)	Healthy	CG:23;EG:22	CG:68.8 ± 4.9;EG:72.1 ± 6.3	No exercise	RT	12	3	60-80% 1-RM	TNF, CRP, IL-6
Kirk et al. (2021) (Kirk et al., 2021)	Healthy	CG:28; EG:22	CG:71.83 ± 6.51; EG:66.63 ± 3.92	No exercise	RT	16	3	60-80% 1-RM	TNF, CRP, IL-6
Strandberg et al. (2015) (Strandberg et al., 2015)	Healthy	CG:18; EG:17	CG:68 ± 1; EG:68 ± 2	No exercise	RT	24	2	75-85% 1-RM	CRP, IL-6
Urzi et al. (2019) (Urzi et al., 2019)	Healthy	CG:9; EG:11	CG:88.9 ± 5.3; EG:88.9 ± 5.3	No exercise	RT	12	3	Borg RPE 12-14	CRP
Gardner et al. (2014) (Gardner et al., 2014)	Cardiovascular Disease	CG:60; EG:60	CG:65 ± 9; EG:67 ± 10	Conventional Treatment	AT	12	3	–	CRP
Beavers et al. (2013) (Beavers et al., 2013)	Obesity	CG:93; EG:97	60-79岁	Health Education	AT	72	5	MET≥3	IL-6
Nicklas et al. (2008) (Nicklas et al., 2008)	Healthy	CG:186; EG:183	CG:77.0 ± 4.4; EG:76.4 ± 4.1	Health Education	MCEx	24	3	Borg 12-16	CRP, IL-6
Miller et al. (2008) (Miller et al., 2008)	Obesity with Arthritis	CG:36; EG:31	CG:69.5 ± 1.0; EG:69.8 ± 1.0	Health Education	MCEx	24	3	50-85% HRR	CRP, IL-6, TNF
Prakhinkit et al. (2014) (Prakhinkit et al., 2014)	Depression	CG:13; EG:14	CG:81.0 ± 1.7; EG:74.8 ± 1.7	No exercise	AT	12	3	40% HRR	CRP, IL-6
Bouchonville et al.(2014) (Bouchonville et al., 2014)	Obesity	CG:27; EG:28	CG:69 ± 4; EG:70 ± 4	No exercise	MCEx	48	3	65-85% HRmax	TNF, CRP
Brooks et al. (2006) (Brooks et al., 2006)	Diabetes Mellitus	CG:32; EG:31	CG:66 ± 1; EG:66 ± 2	Conventional Treatment	RT	16	3	60-80% 1-RM	CRP
Muscari et al.(2010) (Muscari et al., 2010)	Healthy	CG:60;EG:60	CG:69.6 ± 2.8;EG:68.8 ± 2.5	No exercise	AT	52	3	70% HRmax	CRP
Rosa et al.(2025) (Rosa et al., 2025)	Healthy	CG:14;EG:12	CG:69.6 ± 4.6;EG:70.6 ± 4.7	Placebo	RT	12	2	60% RM	TNF, IL-6
Tomeleri et al.(2023) (Tomeleri et al., 2023)	Healthy	CG:15;EG:15	>60岁	No exercise	RT	12	3	10–15 repetitions	CRP, TNF, IL-6
Nawrat-Szołtysik et al.(2022) (Nawrat-Szołtysik et al., 2022)	Healthy	CG:20;EG:22	CG:70.69 ± 6.96;EG:69.00 ± 6.74	No exercise	Whole-body vibration training	12	2	20Hz, amplitude 2mm	IL-6
Souza et al.(2022) (de Sá Souza et al., 2022)	Sarcopenia	CG:14;EG:14	CG:74.64 ± 7.13;EG:77.42 ± 6.25	Health Education	RT	12	3	75% 1-RM	TNF, IL-6
Morawin et al.(2021) (Morawin et al., 2021)	Healthy	CG:21;EG:23	CG:72.1 ± 4.6;EG:73.6 ± 7.9	Health Education	AT	40	2	Yang-style 24-form Tai Chi	CRP, TNF
Sadjapong et al.(2020) (Sadjapong et al., 2020)	Frailty	CG:32;EG:32	CG:78.87 ± 1.32;EG:76.68 ± 1.14	Conventional Treatment	MCEx	12	3	65% 1-RM	IL-6, CRP
Tait et al.(2025) (Tait et al., 2025)	Healthy	CG:144;EG:155	CG:77.7 ± 7.2;EG:77.2 ± 6.6	Conventional Treatment	MCEx	24	2	10–15 repetitions per set	CRP, TNF, IL-6
Ye et al.(2024) (Ye et al., 2024)	Cognitive Impairment	CG:51;EG:51	CG:65.35 ± 5.15;EG:67.68 ± 5.19	Routine Activities	AT	24	3	Moderate Intensity	IL-6, TNF
Griffen et al.(2024) (Griffen et al., 2024)	Healthy	CG:18;EG:18	CG:67 ± 4;EG:67 ± 4	No exercise	RT	12	2	80%1-RM	CRP, TNF, IL-6
Gargallo et al.(2024) (Gargallo et al., 2024)	Metabolic Syndrome	CG:25;EG1:25;EG2:22	CG:68.00 ± 5.01;EG1:69.30 ± 4.96;EG2:67.24 ± 4.42	No exercise	EG1:MCEx;EG2:RT	20	2	Borg 11-15	CRP
Timon et al.(2021) (Timon et al., 2021)	Healthy	CG:19;EG:18	CG:70.5 ± 4.0;EG:70.3 ± 3.3	Routine Activities	RT	24	3	RPE 6-8	CRP, IL-6
Rodrigues-Krause et al.(2018) (Rodrigues-Krause et al., 2018)	Healthy	CG:10;EG:10	66 ± 3岁	Stretching	AT	8	3	60% VO2max	CRP, TNF
Chen et al.(2018) (Chen et al., 2018)	Sarcopenia	CG:16;EG:17	CG:68.3 ± 2.8;EG:66.7 ± 5.3	Routine Activities	RT	8	2	60-70% 1-RM	CRP, TNF, IL-6
Tomeleri et al.(2016) (Tomeleri et al., 2016)	Obesity	CG:19;EG:19	CG:69.5 ± 4.7;EG:66.8 ± 3.2	No exercise	RT	8	3	10–15 Repetition Maximum	CRP, TNF, IL-6
Ribeiro et al.(2016) (Ribeiro et al., 2016)	Healthy	EG1:14;EG2:15	67.6 ± 5.1	No exercise	RT	8	3	60-70% 1-RM	CRP
Wanderley et al.(2013) (Wanderley et al., 2013)	Healthy	CG:19;EG1:11;EG2:20	CG:67.8 ± 5.5;EG1:67.3 ± 4.9;EG2:69.9 ± 5.7	No exercise	EG1:RT;EG2:AT	32	3	RT:80%1RM; AT:70-80%HRR	CRP, TNF, IL-6
Miller et al.(2017) (Miller et al., 2017)	Diabetes Mellitus	CG:13;EG:16	CG:66.9 ± 5.3;EG:67.6 ± 5.2	Weight Loss Training	RT	52	3	75-85%1-RM	TNF
Martins et al.(2010) (Martins et al., 2010a)	Healthy	CG:13;EG1:18;EG2:14	CG:81.2 ± 7.9;EG1:76.2 ± 7.4;EG2:73.2 ± 6.5	No exercise	EG1:AT; EG2:RT	16	3	AT:71-85%HRR; RT:60-70% 1-RM	CRP
Jamrasi et al.(2025) (Jamrasi et al., 2025)	Healthy	CG:27;EG1:27;EG2:27	CG:74.30 ± 5.87;EG1:73.85 ± 4.20;EG2:73.04 ± 4.63	Routine Activities	EG1:RT;EG2:AT+RT	12	3	Moderate Intensity	CRP, TNF, IL-6
Nabuco et al.(2019) (Nabuco et al., 2019)	Sarcopenia	CG:13;EG:13	CG:70.1 ± 3.9;EG:68.0 ± 4.2	Placebo	RT	12	3	12 Repetition Maximum	CRP, TNF, IL-6
Despeghel et al.(2021) (Despeghel et al., 2021)	Healthy	CG:10;EG:30	CG:69.8 ± 4.4;EG:70.4 ± 5.3	No exercise	AT+RT	6	2	60% 1-RM	IL-6, TNF
Eleuteri et al.(2013) (Eleuteri et al., 2013)	CHF	CG:10;EG:11	CG:63 ± 2;EG:66 ± 2	Routine Activities	AT	12	5	Ventilatory Threshold Intensity	IL-6, CRP
Lustosa et al.(2013) (Lustosa et al., 2013)	Healthy	CG:16;EG:32	>65岁	Conventional Treatment	RT	10	3	75% 1-RM	IL-6
Tew et al.(2012) (Tew et al., 2012)	AAA	CG:14;EG:11	CG:74 ± 6;EG:71 ± 8	Conventional Treatment	AT	12	3	Borg 12-14	CRP
Heo et al.(2023) (Heo and Jee, 2024)	Sarcopenia	CG:20;EG1:21;EG2:20;EG3:20	CG:67.30 ± 3.45;EG1:67.81 ± 4.85;EG2:67.45 ± 4.70;EG3:67.95 ± 5.05	No exercise	RT	12	3	6-12 1-RM	TNF, IL-6
Nishida et al.(2015) (Nishida et al., 2015)	Healthy	CG:31;EG:31	CG:69.7 ± 6.6;EG:70.4 ± 5.8	Conventional Treatment	AT	12	3	Cadence: 40–70 steps/minute	TNF, CRP
Chupel et al.(2018) (Chupel et al., 2018)	Healthy	CG:12;EG:13	CG:82 ± 7.5;EG:83.5 ± 7.3	Conventional Treatment	MCEx	14	2	PES 5-7	TNF, IL-6
Su et al.(2022) (Su et al., 2022)	Diabetes Mellitus	CG:13;EG:14	CG:63.61 ± 2.56;EG:64.01 ± 1.98	Conventional Treatment	AT+RT	12	3	AT:65-70%HRmax; RT:70-85%1RM	TNF, IL-6, CRP
Irwin et al.(2012) (Irwin and Olmstead, 2012)	Healthy	CG:37;EG:46	CG:71.4 ± 7.7;EG:70.7 ± 5.9	Health Education	AT	16	3	Moderate Intensity	IL-6, CRP
Rondanelli et al.(2016) (Rondanelli et al., 2016)	Sarcopenia	CG:61;EG:69	CG:80.21 ± 8.54;EG:80.77 ± 6.29	Placebo	MCEx	12	5	Borg 12-14	CRP
Nicklas et al.(2004) (Nicklas et al., 2004)	Obesity with Arthritis	CG:60;EG:53	69 ± 6岁	Health Education	AT+RT	72	3	Moderate Intensity	CRP, TNF, IL-6
Lima et al.(2015) (Lima et al., 2015)	Hypertension	CG:14;EG1:15;EG2:15	CG:69.9 ± 5.5;EG1:67.8 ± 4.3;EG2:67.8 ± 5.2	No exercise	EG1:AT; EG2:AT+RT	8	3	AT:40-70%HRR; RT: Borg 10-13	TNF, IL-6
So et al.(2013) (So et al., 2013)	Healthy	CG:22;EG:18	CG:68.4 ± 5.8;EG:71.6 ± 5.5	No exercise	RT	12	3	–	TNF, IL-6, CRP
Chang et al.(2023) (Chang et al., 2023)	Sarcopenia	CG:57;EG:57	CG:75.00 ± 5.87;EG:75.02 ± 5.91	No exercise	AT+RT	12	2	Moderate Intensity	TNF, IL-6
AbdEl-Kader et al.(2019) (Abd El-Kader and Al-Jiffri, 2019)	Healthy	CG:25;EG:25	CG:65.72 ± 3.86;EG:64.98 ± 4.15	Routine Activities	AT	24	3	60-80% HRmax	TNF;IL-6
AbdEl-Kader et al.(2018) (Abd El-Kader and Al-Shreef, 2018)	Healthy	CG:40;EG:40	CG:66.43 ± 3.71;EG:65.96 ± 3.42	Routine Activities	EG1:AT;EG2:RT	24	3	AT:70-80%HRmax; RT:60-80% 1-RM	TNF;IL-6
Chupel et al.(2017) (Chupel et al., 2017)	Cognitive Impairment	CG:16;EG:16	CG:82.12 ± 6.41;EG:83.50 ± 5.13	No exercise	RT	28	2—3	6–8 PSE	CRP
Andersson et al.(2020) (Andersson et al., 2020)	RA	CG:24;EG:25	CG:70.0 ± 2.4;EG:69.0 ± 2.7	Routine Activities	MCEx	20	3	HRmax70-89%	CRP
Beltran et al.(2014) (Beltran Valls et al., 2014)	Healthy	CG:13;EG:11	CG:72.0 ± 3.16;EG:72.0 ± 3.65	No exercise	RT	12	2	70% 1-RM	CRP
Byrkjeland et al.(2011) (Byrkjeland et al., 2011)	CHF	CG:40;EG:40	CG:71.5 ± 7.8;EG:68.8 ± 7.9	Conventional Treatment	AT	16	2	11–13 Borg	CRP;TNF;IL-6
Kim et al.(2019) (Kim et al., 2019)	Hypertension	CG:18;EG:18	CG:71.72 ± 4.56;EG:72.67 ± 4.02	No exercise	AT	12	3	60-80% HRR	TNF;CRP
Gómez-Tomás et al.(2018) (Gómez-Tomás et al., 2018)	Healthy	CG:20;EG:18	CG:70.45 ± 5.44;EG:70.89 ± 4.42	No exercise	RT	48	3	4-7O MNI-RES	CRP
Ihalainen et al.(2019) (Ihalainen et al., 2019)	Healthy	CG:20;EG:74	CG:69.4 ± 2.2;EG:69.5	No exercise	RT	24	1—3	70-90%1-RM	IL-6;CRP
Jensen et al.(2019) (Jensen et al., 2019)	AD	CG:92;EG:106	CG:71.4 ± 7.3;EG:69.9 ± 7.5	Conventional Treatment	AT	16	3	Moderate Intensity	TNF;IL-6
Kato et al.(2019) (Kato et al., 2019)	Cardiovascular Disease	CG:31;EG:28	CG:65.0 ± 8.0;EG:67.0 ± 10.0	Conventional Treatment	AT+RT	24	2—3	RT:40-60%1RM	IL-6;CRP
Kim et al.(2018) (Kim et al., 2018)	Healthy	CG:10;EG:25	CG:73.2 ± 4.9;EG:73.2 ± 4.9	No exercise	EG1:RT;EG2:AT+RT	6	3	RT:8-12RM; AT+RT: Moderate Intensity	IL-6
Luk et al.(2012) (Luk et al., 2012)	CHD	CG:32;EG:21	CG:66.6 ± 7.9;EG:67.7 ± 9.0	Conventional Treatment	AT+RT	8	3	80% HRmax	CRP
Petersen et al.(2008) (Petersen et al., 2008)	Stable COPD	CG:9;EG:9	CG:66.0 ± 9.48;EG:67.0 ± 9.0	Conventional Treatment	AT	7	2	85% HRmax	IL-6;TNF;CRP
Forti et al.(2014) (Forti et al., 2014)	Healthy	CG:20;EG:20	CG:67.7 ± 7.4;EG:66.4 ± 9.1	No exercise	RT	12	3	70–80% 1-RM	IL-6
Rodriguez-Miguelez et al.(2014) (Rodriguez-Miguelez et al., 2014)	Healthy	CG:10;EG:16	CG:69.1 ± 1.1;EG:70.0 ± 0.9	No exercise	RT	8	2	60-80% 1-RM	CRP, TNF, IL-6
Ruiz et al.(2015) (Ruiz et al., 2015)	Healthy	CG:20;EG:20	CG:92.1 ± 2.3;EG:92.3 ± 2.3	No exercise	RT	8	3	10–12 Borg	TNF
Beavers et al.(2010) (Beavers et al., 2010)	Healthy	CG:186;EG:182	CG:77.0 ± 4.4;EG:76.4 ± 4.1	Health Education	MCEx	24	3	AT:12–13 Borg; RT:15–16 Borg	IL-6, CRP
Wang et al.(2015) (Wang et al., 2015)	Healthy	CG:20;EG:25	CG:63.39 ± 3.43;EG:63.53 ± 3.12	No exercise	AT	24	5	–	IL-6, TNF
Chen et al.(2020) (Chen et al., 2020)	Healthy	CG:10;EG:20	CG:63.59 ± 2.21;EG:64.75 ± 2.89	No exercise	AR	48	6	12–14 RPE	IL-6, CRP
Ferreira et al.(2018) (Ferreira et al., 2018)	Frailty	CG:24;EG:13	CG:77.8 ± 8.0;EG:73.3 ± 6.4	No exercise	MCEx	12	3	Moderate Intensity	CRP, IL-6
Zhang et al.(2025) (Zhang et al., 2025)	Sarcopenia	CG:60;EG:60	CG:73.2 ± 4.6;EG:72.7 ± 4.8	Conventional Treatment	AT	12	5	60%HRmax, 10-13RPE	TNF, IL-6
Luo et al.(2021) (Luo, 2021)	Type 2 Diabetes Mellitus with Hypertension	CG:40;EG:40	CG:67.32 ± 9.46;EG:66.54 ± 10.32	Conventional Treatment	AT	12	3	11–13 Borg	CRP, TNF, IL-6
Han et al.(2024) (Han et al., 2024)	Sarcopenia	CG:50;EG:50	CG:73.52 ± 2.37;EG:74.18 ± 2.13	Conventional Treatment	RT	4	3	8–12 Repetition Maximum	IL-6, CRP

COPD, Chronic Obstructive Pulmonary Disease; CG, Control Group; EG, Experimental Group; HRmax, Maximal Heart Rate; HRR, Heart Rate Reserve; Borg, Borg Rating of Perceived Exertion Scale; 1-RM, One-Repetition Maximum; RPE, Rating of Perceived Exertion; VO_2_ max, Maximal Oxygen Uptake; OMNI-RES, OMNI Resistance Exercise Scale; CRP, C-Reactive Protein; TNF-α, Tumor Necrosis Factor-alpha; IL-6, Interleukin-6; AT, Aerobic Training; RT, Resistance Training; MCEx, Multicomponent Exercise; MET, Metabolic Equivalent of Task; CHF, Chronic Heart Failure; AAA, Abdominal Aortic Aneurysm; RA, Rheumatoid Arthritis; CHD, Coronary Heart Disease.

### 3.3 Risk of bias

The risk of bias assessment results for the 82 included studies are summarized in **Figure 2**. Based on the Cochrane ‘Risk of Bias’ tool and applying the “shortest plank” principle, the overall methodological quality of the included literature was determined. The results indicate a widespread presence of unclear or high risk of bias. Specifically, only 14 studies were judged to have an overall low risk of bias. The majority of studies (53 studies) were judged to have an overall high risk of bias, primarily due to issues related to blinding. The remaining 15 studies were rated as having an overall unclear risk of bias.

**Figure 2:**
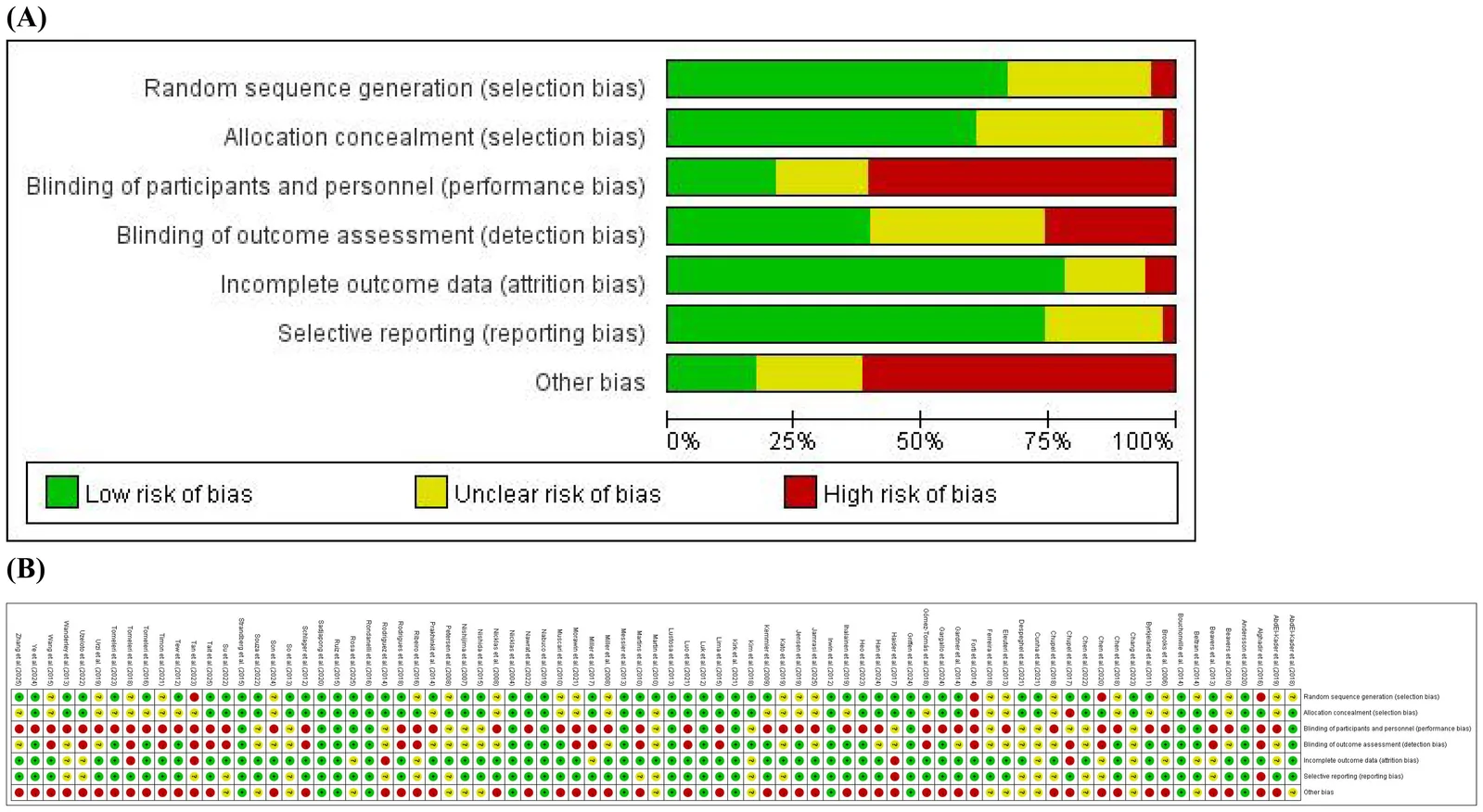
Risk of bias assessment for the 82 included randomized controlled trials using the cochrane risk of bias tool (version 1). **(A)** Risk of bias graph showing the proportion of studies with low, unclear, and high risk of bias across six domains. **(B)** Risk of bias summary displaying individual study-level judgments. The majority of studies (53/82, 64.6%) were rated as having an overall high risk of bias, primarily due to lack of blinding. Only 14 studies (17.1%) were judged as low risk.

### 3.4 Multilevel meta-analysis

Among the included RCTs, 67 studies analyzed the effect of exercise intervention on improving serum IL-6 concentration in the elderly, including a total of 2230 participants in the control group and 2472 participants in the experimental group. A multilevel meta-analysis was performed to obtain the overall effect size (see **Figure 3A**): SMD=-0.31 (95%CI: -0.47, -0.16), *p*<0.001, with a statistically significant difference, indicating that exercise intervention can reduce serum IL-6 concentration in the elderly. However, this result had high heterogeneity (*I*²=82%), which may be caused by differences in the physical status of participants and training methods. Therefore, this study conducted further subgroup analysis based on different training methods. The results showed that compared with MCEx [SMD = 0.06 (95%CI: -0.16, 0.28), *p* = 0.585], RT [SMD=-0.35 (95%CI: -0.62, -0.08), *p* = 0.011], combined training [SMD=-0.45 (95%CI: -0.76, -0.14), *p* = 0.004], and AT [SMD=-0.42 (95%CI: -0.81, -0.03), *p* = 0.033] all had statistically significant reducing effects, indicating that RT, AT and combined training can all effectively improve serum IL-6 concentration in the elderly.

**Figure 3:**
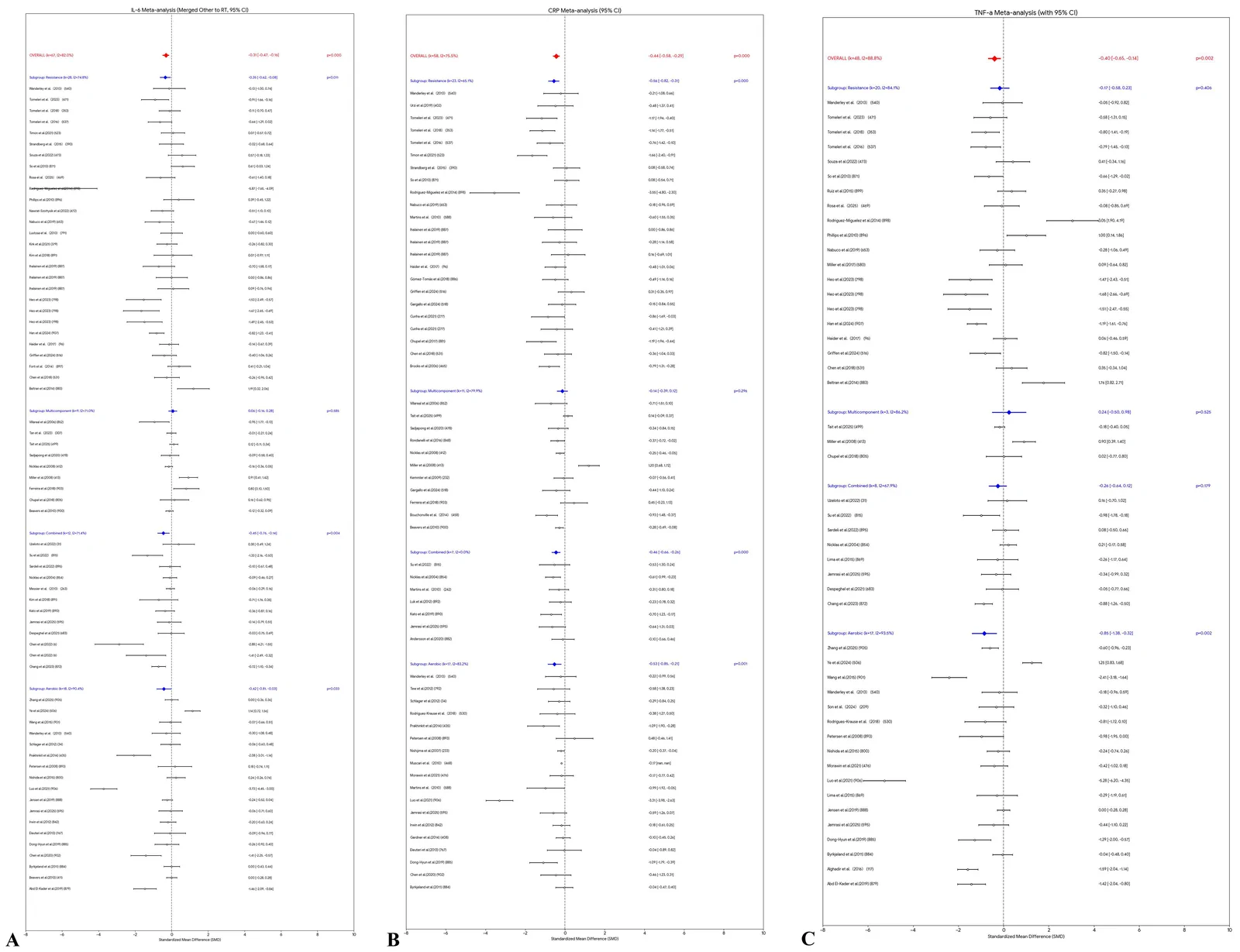
Forest plot of the multilevel meta-analysis comparing the effects of different exercise modalities (AT, RT, AT+RT, MCEx) versus control (CON) on serum inflammatory biomarkers in older adults. Effect sizes are presented as standardized mean differences (SMD) with 95% confidence intervals (95% CI). Negative SMD values favor exercise intervention (reduction in inflammatory marker levels). The size of each square is proportional to the study weight. The diamond represents the pooled effect size for each exercise modality. Heterogeneity is reported as I² statistics. **(A)** Interleukin-6 (IL-6): 67 studies, total I² = 82.0%. **(B)** C-reactive protein (CRP): 58 studies, total I² = 75.5%. **(C)** Tumor necrosis factor-α (TNF-α): 48 studies, total I² = 88.8%. AT, aerobic training; RT, resistance training; AT+RT, combined training; MCEx, multicomponent exercise; CON, control.

58 studies analyzed the effect of exercise intervention on improving serum CRP concentration in the elderly, including a total of 1913 participants in the control group and 2085 participants in the experimental group. A multilevel meta-analysis was performed to obtain the overall effect size (see **Figure 3B**): SMD=-0.44 (95%CI: -0.58, -0.29), *p*<0.001, with a statistically significant difference, indicating that exercise intervention can reduce serum CRP concentration in the elderly. However, this result had high heterogeneity (I²=75.5%). Further subgroup analysis was conducted based on different training methods. The results showed that compared with multicomponent training [SMD=-0.14 (95%CI: -0.39, 0.12), *p* = 0.296], resistance training [SMD=-0.56 (95%CI: -0.82, -0.31), *p*<0.001], combined training [SMD=-0.46 (95%CI: -0.66, -0.26), *p*<0.001], and aerobic training [SMD=-0.53 (95%CI: -0.85, -0.21), *p* = 0.001] all had statistically significant reducing effects, indicating that resistance training, aerobic training, and combined training can all effectively improve serum CRP concentration in the elderly.

48 studies analyzed the effect of exercise intervention on improving serum TNF-α concentration in the elderly, including a total of 1425 participants in the control group and 1584 participants in the experimental group. A multilevel meta-analysis was performed to obtain the overall effect size (see **Figure 3C**): SMD=-0.40 (95%CI: -0.65, -0.14), *p* = 0.002, with a statistically significant difference, indicating that exercise intervention can reduce serum TNF-α concentration in the elderly. However, this result had high heterogeneity (I²=88.8%). Further subgroup analysis was conducted based on different training methods. The results showed that among the four exercise intervention methods, only aerobic training [SMD=-0.85 (95%CI: -1.38, -0.32), *p* = 0.002] had a statistically significant reducing effect, suggesting that compared with other training methods, aerobic training may have a better effect on improving serum TNF-α concentration in the elderly.

To further explore the intervention effects of different exercise intervention methods on serum IL-6, CRP, TNF-αlevels in elderly individuals with different physical statuses, this study conducted a double subgroup analysis based on different exercise intervention methods and different physical statuses of the elderly (**Table 5**). The results showed that for elderly individuals in a healthy state, none of the four intervention methods (AT, RT, combined training, and MCEx) had a statistically significant effect on improving IL-6 levels. For the elderly population with chronic diseases, both RT [SMD=-0.685 (95%CI: -1.163, -0.207), *p* = 0.005] and AT+RT [SMD=-0.531 (95%CI: -0.913, -0.150), *p* = 0.006] had statistically significant effects on improving IL-6 levels, while AT and MCEx had no statistically significant improvement effects.

**Table 5:** Multilevel meta-analysis results of the effects of different exercise modalities on IL-6 levels in older adults with healthy and chronic disease status.

Outcome	Subject status	Exercise modality	n	SMD	95%CI	*p*	I^2^
IL-6	Healthy	AT	331	-0.354	(-0.741, 0.033)	0.074	75.913
		RT	405	-0.197	(-0.533, 0.139)	0.251	74.196
		AT+RT	70	-0.197	(-0.636, 0.247)	0.381	0
		MCEx	553	-0.056	(-0.196, 0.086)	0.448	20.735
	Chronic Disease	AT	376	-0.478	(-1.137, 0.180)	0.155	93.948
		RT	190	-0.685	(-1.163, -0.207)	0.005	72.769
		AT+RT	365	-0.531	(-0.913, -0.150)	0.006	78.491
		MCEx	180	0.161	(-0.358, 0.681)	0.543	81.330
CRP	Healthy	AT	224	-0.347	(-0.712, 0.0189)	0.062	41.536
		RT	311	-0.591	(-0.945, -0.238)	0.001	71.853
		AT+RT	59	-0.425	(-0.822, -0.029)	0.036	0
		MCEx	144	-0.295	(-0.579, -0.012)	0.041	77.099
	Chronic Disease	AT	511	-0.618	(-1.120, -0.116)	0.016	90.220
		RT	134	-0.541	(-0.831, -0.249)	<0.001	19.122
		AT+RT	141	-0.467	(-0.698, -0.236)	<0.001	0
		MCEx	203	0.063	(-0.449, 0.575)	0.809	83.288
TNF-α	Healthy	AT	265	-0.872	(-1.369, -0.375)	<0.001	81.387
		RT	254	0.147	(-0.449, 0.745)	0.629	85.813
		AT+RT	57	-0.207	(-0.691, 0.277)	0.402	0
		MCEx	168	-0.161	(-0.379, 0.057)	0.147	0
	Chronic Disease	AT	339	-0.847	(-1.744, 0.051)	0.065	96.076
		RT	186	-0.537	(-1.078, 0.005)	0.052	81.177
		AT+RT	177	-0.278	(-0.770, 0.215)	0.269	76.654
		MCEx	118	0.899	(0.395, 1.402)	0.001	0

AT, aerobic training; RT, resistance training; AT+RT, combined training; MCEx, multicomponent exercise; IL-6, interleukin-6; CRP, C-reactive protein; TNF-α, tumor necrosis factor-alpha. The “n” column represents the total number of participants in the exercise group across studies contributing to that specific subgroup analysis.

For elderly individuals in a healthy state, both RT [SMD=-0.591 (95%CI: -0.945, -0.238), *p* = 0.001] and combined training [SMD=-0.425 (95%CI: -0.822, -0.029), *p* = 0.036] had statistically significant effects on improving CRP levels. For the elderly population with chronic diseases, except for MCEx [SMD = 0.063 (95%CI: -0.449, 0.575), *p* = 0.809], AT [SMD=-0.618 (95%CI: -1.120, -0.116), p=0.016], RT [SMD=-0.541 (95%CI: -0.831, -0.249), *p*<0.001], and combined training [SMD=-0.467 (95%CI: -0.698, -0.236), *p*<0.001] all had statistically significant effects on improving CRP levels.

For elderly individuals in a healthy state, aerobic training (AT) [SMD=-0.872 (95%CI: -1.369, -0.375), *p*<0.001] had a statistically significant effect on improving TNF-α levels; for the elderly population with chronic diseases, none of the four training methods could effectively reduce the TNF-α levels of the subjects.

Subgroup difference tests. For IL-6, health status did not significantly modify the effect (*p* = 0.82), whereas exercise modality showed a trend toward significance (*p* = 0.06). For CRP, health status was not a significant modifier (*p* = 0.12), but exercise modality significantly influenced the effect size (*p* = 0.008). For TNF-α, neither health status (*p* = 0.47) nor exercise modality (*p* = 0.76) showed statistically significant subgroup differences.

For IL-6, publication bias was assessed using the rank correlation test (Kendall’s τ) adapted for multilevel models and Egger’s regression test. The rank correlation test suggested the potential presence of small-study effects or publication bias (Kendall’s τ = -0.460, *p* < 0.001); Egger’s test also showed significance (intercept = -1.95, *p* = 0.0049). The funnel plot (see **Figure 4A**) exhibited visual asymmetry, which was consistent with the above test results, further supporting the possibility of small-study effects or publication bias.

**Figure 4:**
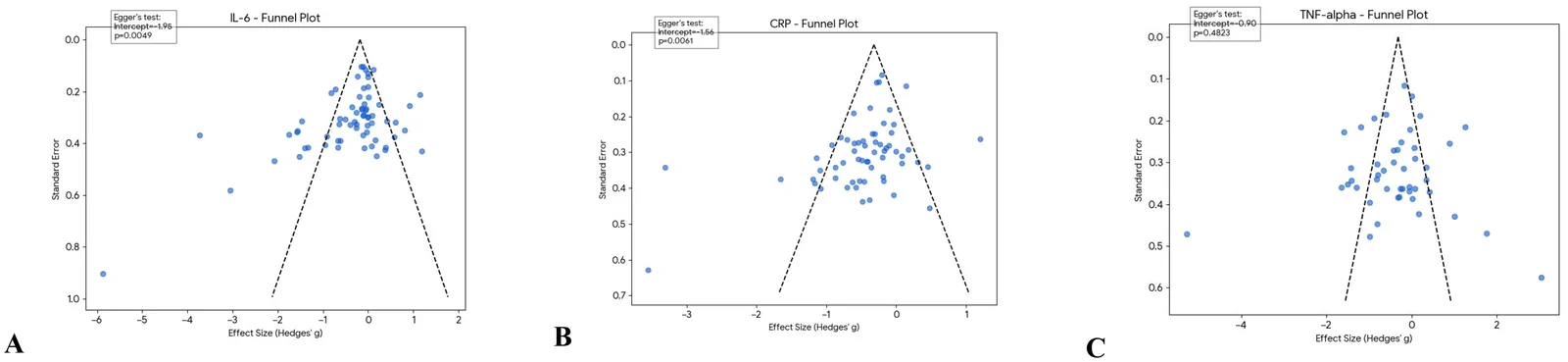
Funnel plot assessing publication bias for the meta-analysis of exercise interventions on IL-6, CRP, TNF-α levels in older adults. Each plot shows the standardized mean difference (SMD) on the x-axis against the standard error (SE) on the y-axis. The vertical line represents the pooled effect estimate. In the absence of publication bias, studies should be symmetrically distributed around the pooled effect. **(A)** IL-6. **(B)** CRP. **(C)** TNF-α.

For CRP, the rank correlation test suggested the potential presence of small-study effects or publication bias (Kendall’s τ = -0.491, p < 0.001); Egger’s test also showed significance (intercept = -1.56, p = 0.0061). The funnel plot (see **Figure 4B**) exhibited visual asymmetry, which was consistent with the above test results.

For TNF-α, the rank correlation test suggested the potential presence of small-study effects or publication bias (Kendall’s τ = -0.491, *p* < 0.001); the result of Egger’s test was not significant (intercept = -0.90, *p* = 0.4823). The funnel plot (see **Figure 4C**) exhibited a certain degree of visual asymmetry, but the Egger’s test did not reach a significant level, suggesting a low possibility of publication bias.

Trim-and-fill analysis (**Supplementary Figure 1**) imputed 11 missing studies for IL-6, 11 for CRP, and 13 for TNF-α. The adjusted random-effects effect sizes were modestly larger in magnitude than the original estimates (IL-6: g = −0.531; CRP: g = −0.583; TNF-α: g = −0.904), with all 95% confidence intervals excluding zero, indicating that publication bias does not reverse the overall conclusions.

To assess the robustness of the pooled effect size results in this study, two sensitivity analyses were sequentially conducted, and the specific results are as follows:

First, a leave-one-out sensitivity analysis was performed, where the pooled effect size was recalculated after sequentially removing each included study. The results showed that for IL-6, the range of the pooled effect size was between -0.34 and -0.33 (see **Figure 5A**), which only slightly deviated from the primary analysis result (SMD = -0.331), indicating that no single study exerted an excessive impact on the overall effect size, i.e., the removal of a single study would not change the conclusion of the overall analysis. For CRP, the range of the pooled effect size was between -0.95 and -0.48 (see **Figure 5B**), which did not fundamentally change compared with the primary analysis result (SMD = -0.448), and all estimates showed a negative effect direction, suggesting that no single study had a decisive impact on the overall effect. For TNF-α, after sequentially removing any single study, the range of the pooled effect size was between -0.39 and 0.36 (see **Figure 5C**), which fluctuated to a certain extent compared with the primary analysis result (SMD = -0.39), and the direction of the effect size changed after removing some studies, indicating that individual studies may have a significant impact on the overall effect.

**Figure 5:**
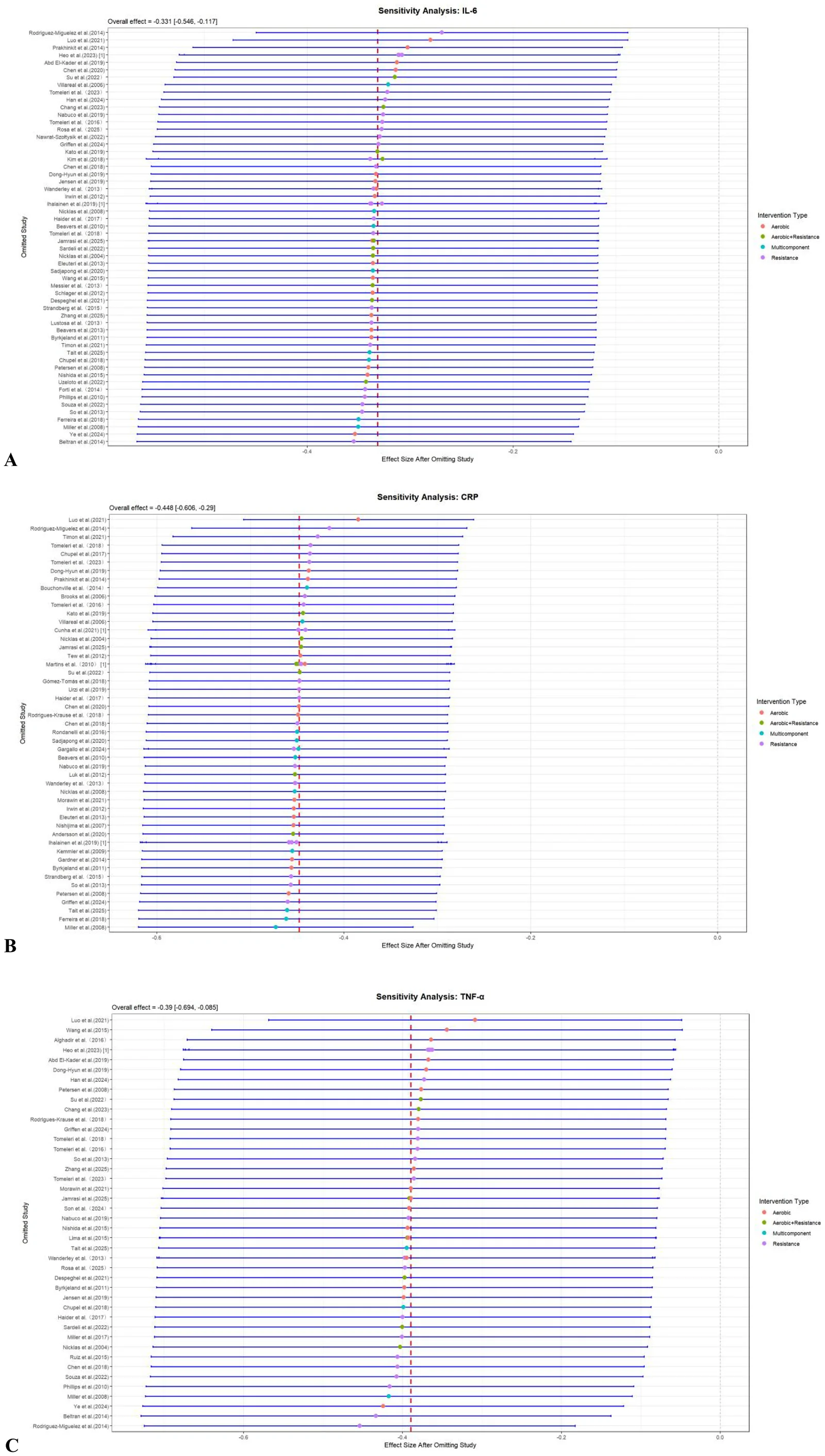
Leave-one-out sensitivity analysis for **(A)** IL-6, **(B)** CRP, and **(C)** TNF-α. Each panel shows the effect size when each study is omitted, with studies color-coded by intervention type (Aerobic, Aerobic+Resistance, Multicomponent, Resistance). Vertical dashed lines represent the overall effect estimates.

Second, considering that a single study might contribute multiple effect sizes in the multilevel model, leading to the issue of effect size correlation, this study further conducted a sensitivity analysis by adjusting the intraclass correlation coefficient (ICC, rho). The multilevel random-effects model was refitted with rho values set to 0, 0.3, and 0.5, respectively, and the specific results are as follows:

For IL-6, when rho = 0, SMD = -0.331 (95% CI: -0.536, -0.117); when rho = 0.3, SMD = -0.377 (95% CI: -0.656, -0.098); when rho = 0.5, SMD = -0.377 (95% CI: -0.656, -0.097). Compared with rho = 0 set in the primary analysis, the effect size estimates under different rho values only changed extremely slightly, and the direction and statistical significance of all effect size estimates remained consistent (95% confidence intervals did not include 0).

For CRP, when rho = 0, SMD = -0.448 (95% CI: -0.606, -0.29); when rho = 0.3, SMD = -0.452 (95% CI: -0.625, -0.279); when rho = 0.5, SMD = -0.455 (95% CI: -0.630, -0.279). Compared with the primary analysis result (rho = 0), the effect size estimates changed minimally, and the confidence intervals of all estimates did not include 0, with consistent statistical significance.

For TNF-α, when rho = 0, SMD = -0.39 (95% CI: -0.694, -0.085); when rho = 0.3, SMD = -0.402 (95% CI: -0.764, -0.039); when rho = 0.5, SMD = -0.402 (95% CI: -0.765, -0.039). Compared with the primary analysis result (rho = 0), the effect size estimates changed slightly, and the confidence intervals of all estimates did not include 0, with consistent statistical significance.

### 3.5 Network meta-analysis

The network diagram (see **Figure 6**) reflects the comparative relationships between various intervention measures in the included studies. The circles in the figure represent different intervention measures, and the diameter of the circles is positively correlated with the total sample size of participants included in the intervention measure. The lines represent the existence of direct comparisons between two intervention measures (i.e., at least one study directly compared these two measures), and the thickness of the lines is positively correlated with the number of studies for direct comparison. This network meta-analysis mainly analyzed three outcome indicators: IL-6, CRP, and TNF-α, aiming to evaluate the effects of the four included exercise intervention measures (AT, RT, AT+RT, and MCEx) on inflammatory factors in the elderly. First, for IL-6, a total of 67 studies were included in this study, including 2230 participants in the control group and 2472 participants in the experimental group. Among these studies, 28 studies analyzed RT, 18 studies analyzed AT, 12 studies adopted the AT+RT exercise intervention method, and 9 studies investigated the intervention effect of MCEx. Second, for CRP, a total of 58 studies were included in this study, including 1913 participants in the control group and 2085 participants in the experimental group. Among these studies, 23 studies analyzed RT, 11 studies adopted MCEx as the exercise intervention method, 7 studies investigated the intervention effect of AT+RT, and 17 studies analyzed AT. Finally, for TNF-α, a total of 48 studies were included in this study, including 1425 participants in the control group and 1584 participants in the experimental group. Among these studies, 20 studies adopted RT as the exercise intervention method, 17 studies adopted AT, 8 studies analyzed the intervention effect of AT+RT, and 3 studies investigated the intervention effect of MCEx.

**Figure 6:**
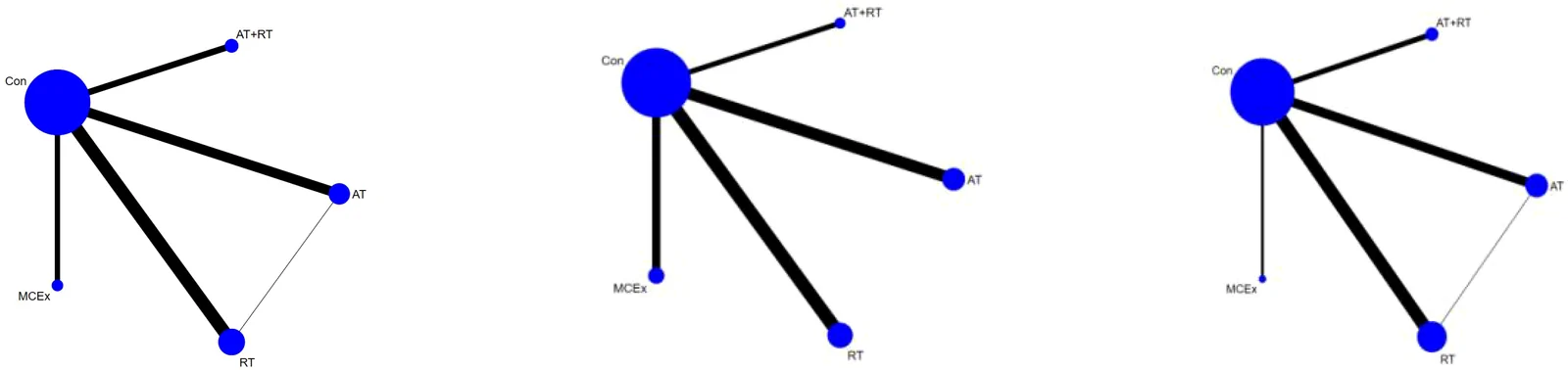
Network of evidence for comparisons of different exercise modalities on inflammatory biomarker levels in older adults. This title is intended for three separate network meta-analysis plots. From left to right, they should represent the evidence networks for IL-6, CRP, and TNF-α, respectively.Each node represents an intervention (AT, aerobic training; RT, resistance training; AT+RT, combined training; MCEx, multicomponent exercise; CON, control). Node size is proportional to the total number of participants randomized to that intervention. Edge thickness is proportional to the number of direct comparison studies between two interventions.

The inconsistency test for IL-6 and TNF-α showed that all p-values were > 0.05 (IL-6: 0.3446; TNF-α: 0.8488), indicating that no significant inconsistency was found in the network. Meanwhile, the node-splitting method showed that all p-values between direct and indirect comparisons of various exercise intervention measures for the three outcome indicators were greater than 0.05 (see **Table 6**), indicating a low possibility of local inconsistency. Therefore, a consistency model was used for this network meta-analysis.

**Table 6:** Results of node-splitting method for testing inconsistency in network meta-analysis.

Outcome	Side	Direct		Indirect		Difference			p-value
		Coef.	Std. err.	Coef.	Std. err.	Coef.	Std. err.	P>z	
IL-6	A C	0.5106875	0.2279092	1.308725	0.8380827	-0.7980377	0.8684472	0.358	0.7941202
	A E	1.005803	0.8293416	0.174222	0.2941505	0.8315806	0.8799614	0.345	0.7944646
	B C *	0.3992187	0.2586427	1.123528	19.07908	-0.7243093	19.08089	0.97	0.7930548
	C D *	-0.0231645	0.315265	-1.096419	23.91788	1.073255	23.91999	0.964	0.793052
	C E	-0.3356557	0.1871474	0.4902516	0.8587721	-0.8259073	0.8787231	0.347	0.7944235
CRP	A C *	0.4954722	0.1701062	0.2446474	5.271469	0.2508248	5.27421	0.962	0.5696685
	B C *	0.4410208	0.2399271	1.005427	23.90997	-0.5644065	23.91116	0.981	0.5696604
	C D *	-0.142674	0.1869644	-0.9618713	19.07112	0.8191974	19.07203	0.966	0.5696619
	C E *	-0.6773215	0.1598135	-0.9598081	14.91347	0.2824866	14.91434	0.985	0.5696622
TNF-α	A C	0.9561098	0.2858144	1.12741	1.056758	-0.1712999	1.094938	0.876	1.01496
	A E	1.13385	1.043726	0.9218749	0.383266	0.211975	1.11187	0.849	1.015349
	B C *	0.2522191	0.3720142	1.958503	22.37543	-1.706283	22.37862	0.939	1.000477
	C D *	0.1590006	0.515259	-1.919838	31.63826	2.078839	31.64244	0.948	1.000427
	C E	-0.0314034	0.2554271	0.1688502	1.080034	-0.2002536	1.109961	0.857	1.015315

Under the consistency model, the relative efficacy ranking of each intervention measure was evaluated by the SUCRA value (see **Table 7**). The specific results were as follows: for reducing IL-6 levels, the SUCRA value ranking of each intervention measure was: AT (84.6%) > AT+RT (71.2%) > RT (59.8%) > MCEx (22.7%) > CON (18.1%), among which AT had the highest probability of being the optimal intervention measure (54.7%). For reducing CRP levels, the ranking was: RT (89.1%) > AT (67.2%) > AT+RT (61.0%) > MCEx (26.3%) > CON (6.4%), and RT had the highest probability of being the optimal intervention measure (65.1%). For reducing TNF-α levels, the ranking was: AT (97.5%) > AT+RT (56.8%) > RT (36.4%) > CON (33.3%) > MCEx (26.0%), and the probability of AT being the optimal intervention measure was as high as 90.6%, suggesting that it has an advantage in improving TNF-α levels in the elderly.

**Table 7:** Treatment rankings based on the surface under the cumulative ranking curve (SUCRA) and probability of being the best treatment for reducing IL-6, CRP, and TNF-α levels.

Outcome	Treatment	SUCRA	PrBest	MeanRank
IL-6	AT	84.6	54.7	1.6
	AT+RT	71.2	32.4	2.2
	RT	59.8	11.5	2.6
	MCEx	22.7	0.3	3.7
	Con	18.1	0.0	4.3
CRP	AT	67.2	17	2.3
	AT+RT	61	17.3	2.6
	Con	6.4	0	4.7
	MCEx	26.3	0.6	3.9
	RT	89.1	65.1	1.4
TNF-α	AT	97.5	90.6	1.1
	AT+RT	56.8	6.8	2.7
	Con	33.3	0	3.7
	MCEx	26	2.3	4
	RT	36.4	0.4	3.5

Higher SUCRA values (range 0-100%) indicate a greater likelihood of being the most effective intervention for reducing the respective inflammatory marker. Mean rank ranges from 1 (best) to 5 (worst). Results are derived from the consistency model of the network meta-analysis.

Rank uncertainty. The SUCRA values in **Table 7** provide summary rankings but do not fully capture the uncertainty around each intervention’s rank position. To address this, we generated rank probability plots (rankograms) for each outcome, presented in **Figure 7**. In these plots, each bar represents an intervention, and the colored segments within each bar indicate the probability (0–100%) that the intervention achieves a specific rank (1st = best, 5th = worst). For example, for IL-6 (**Figure 7A**), AT has a 54.7% probability of being ranked 1st (darkest segment) and a 32.5% probability of being ranked 2nd, indicating that while AT is the most likely best intervention, there is considerable uncertainty (∼45% chance that another modality could be superior). In contrast, MCEx shows a high probability of being ranked 4th or 5th. These probability distributions should be considered alongside the pairwise comparison confidence intervals when interpreting treatment rankings.

**Figure 7:**
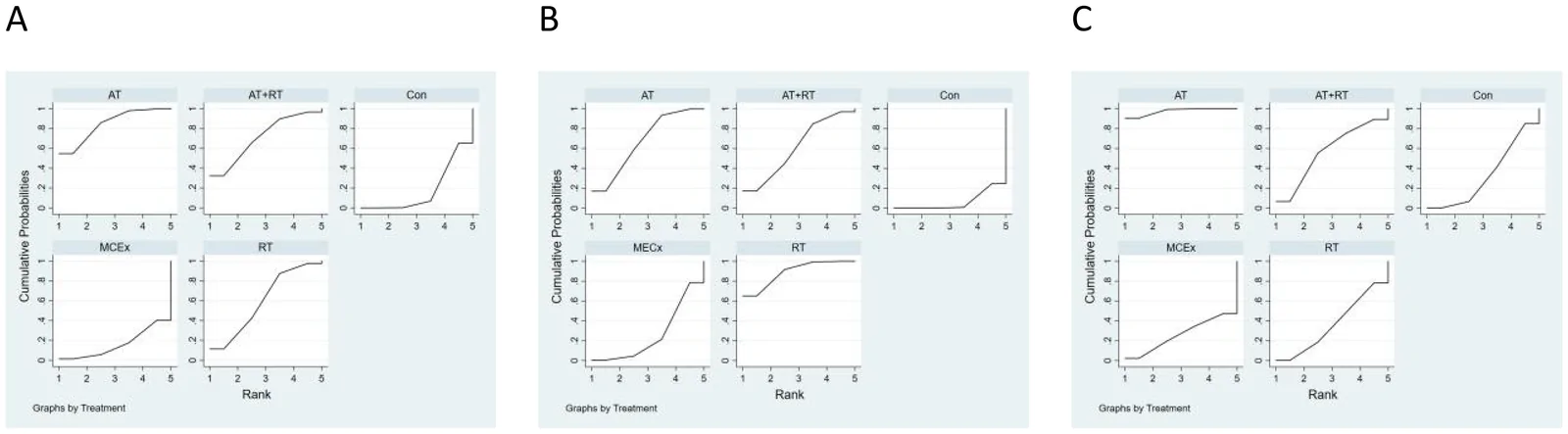
Cumulative probability (SUCRA) curves for the ranking of exercise modalities on inflammatory biomarkers. Cumulative probability curves showing the probability that each intervention achieves a given rank or better (1st = best, 5th = worst) for **(A)** interleukin-6 (IL-6), **(B)** C-reactive protein (CRP), and **(C)** tumor necrosis factor-α (TNF-α).

The pairwise comparison results of the network meta-analysis showed (see **Table 8**) that different exercise modes had different effects on inflammatory markers (IL-6, CRP, TNF-α). Compared with the CON, AT showed statistically significant anti-inflammatory effects on all three outcome indicators: IL-6 (SMD = -0.50, 95% CI: **-**0.87∼**-**0.12), CRP (SMD = -0.50, 95% CI: -0.83∼-0.16), and TNF-α (SMD = -0.97, 95% CI: -1.50∼-0.43). RT significantly reduced CRP levels (SMD = -0.68, 95% CI: -0.99∼-0.36), but its improvement effects on IL-6 and TNF-α did not reach statistical significance. AT+RT showed a positive anti-inflammatory trend in all three outcomes, but did not reach statistical significance. MCEx had no statistically significant differences compared with the control group in all outcomes.

**Table 8:** Pairwise comparison results from the network meta-analysis (standardized mean differences with 95% confidence intervals) for the effects of different exercise modalities on IL-6, CRP, and TNF-α levels in older adults.

Compare	IL-6: SMD (95% CI)	CRP: SMD (95% CI)	TNF-α: SMD (95% CI)
AT vs Con	-0.50 (-0.87, -0.12)	-0.50 (-0.83, -0.16)	-0.97 (-1.50, -0.43)
RT vs Con	-0.29 (-0.63, 0.05)	-0.68 (-0.99, -0.36)	-0.02 (-0.50, 0.46)
AT+RT vs Con	-0.40 (-0.88, 0.09)	-0.44 (-0.91, 0.03)	-0.25 (-0.98, 0.48)
MCEx vs Con	0.08 (-0.44, 0.60)	-0.14 (-0.51, 0.22)	-0.16 (-1.17, 0.85)
AT vs RT	-0.21 (-0.70, 0.29)	0.18 (-0.27, 0.64)	-0.71 (-1.62, 0.19)
AT vs AT+RT	-0.10 (-0.71, 0.51)	-0.05 (-0.63, 0.52)	-0.23 (-1.10, 0.64)
AT vs MCEx	-0.57 (-1.21, 0.07)	-0.35 (-0.85, 0.14)	-0.94 (-1.64, -0.25)
AT+RT vs RT	-0.11 (-0.70, 0.48)	0.24 (-0.33, 0.80)	-0.23 (-1.10, 0.64)
AT+RT vs MCEx	-0.47 (-1.18, 0.23)	-0.30 (-0.89, 0.30)	-0.41 (-1.66, 0.83)
RT vs MCEx	-0.37 (-0.99, 0.25)	-0.53 (-1.02, -0.05)	-0.18 (-1.30, 0.94)

Data are standardized mean differences (SMD) with 95% confidence intervals (95% CI).

In the direct comparisons between intervention measures, AT was significantly superior to MCEx in reducing TNF-α levels (SMD = -0.94, 95% CI:-1.64∼-0.25), and RT was significantly superior to MCEx in reducing CRP levels (SMD = -0.53, 95% CI:-1.02∼-0.05). No statistically significant differences were found between AT and RT, AT and AT+RT, or AT+RT and RT in all outcome indicators, suggesting that these three active intervention measures may have equivalent anti-inflammatory effects. However, it should be noted that the confidence intervals of some comparisons (especially those involving TNF-α) were relatively wide, reflecting a certain degree of uncertainty in the relevant results. In summary, AT may be the most stable and effective exercise mode for reducing systemic inflammation in the elderly, while RT has a more clear improvement effect on CRP levels.

### 3.6 GRADE evidence quality grading

The overall quality of evidence regarding the effects of exercise intervention on serum IL-6, CRP, and TNF-α concentrations in the elderly was assessed using the GRADE framework (see **Table 9**). For all three key outcome indicators, the quality of evidence was rated as very low. This rating indicates that our confidence in the reported effect estimates is limited, and the true effect may be substantially different from the current estimates.

**Table 9:** GRADE evidence profile for outcomes.

Quality assessment							No of patients		Effect			Quality	Importance
No of studies	Design	Risk of bias	Inconsistency	Indirectness	Imprecision	Other considerations	Ex	Control	Relative (95% CI)		Absolute		
Ex for IL-6													
63	randomised trials	serious	serious	no serious indirectness	no serious imprecision	reporting bias strong association	2472	2203	–	SMD 0.331 lower (0.546 to 0.117 lower)		⊕OOO VERY LOW	CRITICAL
Ex for CRP													
60	randomised trials	serious	serious	no serious indirectness	no serious imprecision	reporting bias	2165	2001	–	SMD 0.448 lower (0.606 to 0.29 lower)		⊕OOO VERY LOW	CRITICAL
Ex for TNF-α													
47	randomised trials	serious	serious	no serious indirectness	no serious imprecision	reporting bias	1397	1385	–	SMD 0.39 lower (0.694 to 0.085 lower)		⊕OOO VERY LOW	CRITICAL

Based on the following considerations, the initial high level of evidence from randomized controlled trials was downgraded by three levels for each biomarker. First, it was downgraded by one level due to serious risk of bias. The risk of bias assessment showed that most of the included studies (53 out of 82) had an overall high risk of bias, mainly resulting from insufficient blinding of participants, intervention implementers, and outcome assessors (performance bias and detection bias). This prevalent methodological flaw introduces uncertainty regarding the reliability of the observed effects. Second, it was downgraded by one level due to serious inconsistency. Although the pooled estimates were statistically significant, there was considerable statistical heterogeneity among studies (I² = 82% for IL-6, 75.5% for CRP, and 88.8% for TNF-α). This significant heterogeneity indicates substantial variation in effect sizes, which may be attributed to clinical heterogeneity in participant characteristics (e.g., health status) and methodological heterogeneity in exercise protocols and intervention durations, thereby reducing confidence in the precise pooled estimates. Third, it was downgraded by one level due to suspected publication bias. Assessments using funnel plots and Egger’s test suggested the potential presence of small-study effects or publication bias, particularly for IL-6 and CRP outcomes, indicating that the effect sizes obtained from the meta-analysis may overestimate the true intervention effects.

In summary, although the meta-analysis suggests that exercise intervention is associated with reductions in IL-6 (SMD = -0.331), CRP (SMD = -0.448), and TNF-α (SMD = -0.390), the very low quality of evidence means that these findings must be interpreted with extreme caution. These estimates are likely to change with future research. To strengthen the evidence base, future randomized controlled trials should adopt more rigorous methodologies, especially adequate blinding procedures, and strive for greater consistency and more detailed reporting in terms of intervention protocols and participant characteristics.

## 4 Discussion

This study summarized a large number of RCTs on exercise intervention for chronic inflammatory indicators in the elderly by means of multilevel meta-analysis and network meta-analysis. Among them, the results of multilevel meta-analysis showed that exercise intervention could effectively reduce the levels of IL-6 [SMD=-0.31 (95%CI: -0.47, -0.16), *p*<0.001], CRP [SMD=-0.44 (95%CI: -0.58, -0.29), *p*<0.001] and TNF-α [SMD=-0.40 (95%CI: -0.65, -0.14), *p* = 0.002] in the elderly. These findings are broadly consistent with previous meta-analyses [Khalafi et al., 2023 (Sheldon et al., 2026); Sheldon et al., 2026 (Nejatian Hoseinpour et al., 2025)], but the very low GRADE certainty of the evidence means that the true anti-inflammatory effect may differ substantially from the present estimates. Therefore, all conclusions should be interpreted with caution.

### 4.1 Comparative efficacy of different exercise modalities

Meanwhile, based on different exercise intervention methods, this study conducted relevant subgroup analyses. The results showed that compared with the effect sizes of other exercise intervention methods, AT+RT [SMD=-0.45 (95%CI: -0.76, -0.14), *p* = 0.004] had the best intervention effect on IL-6, RT [SMD=-0.591 (95%CI: -0.945, -0.238), *p* = 0.001] had the best effect on CRP, and AT [SMD=-0.85 (95%CI: -1.38, -0.32), *p* = 0.002] had the best effect on TNF-α. Among them, the meta-analysis results of Khalafi et al. (Sheldon et al., 2026) also indicated that compared with other exercise methods, AT+RT combined training had the best effect on reducing IL-6 levels [SMD=−0.26 (−0.52, −0.005), *p* = 0.04], and AT had the best effect on reducing TNF-α levels [SMD=−0.44, (−0.77, -0.11), *p* = 0.009]. At the same time, the intervention effect of RT on CRP was consistent with the meta-analysis results of Kim et al. (Kim and Yeun, 2022) and Sardeli et al (Cheng and Yang, 2024), both of which believed that resistance training could effectively improve CRP levels in the elderly [SMD=−0.64, (−1.02, 0.26), *p*<0.001], [SMD=-0.72, (-1.06, -0.38), *p*<0.001]. However, in comparison, Khalafi et al. only included 6 RCTs in their analysis of AT+RT intervention on IL-6 and AT intervention on TNF-α, respectively, Sardeli et al. only included 8 RCTs in their analysis of RT intervention on CRP, and Kim et al. did not conduct correlation processing on the multi-arm trials in the included studies when conducting the meta-analysis, so their results may have a risk of bias. Therefore, compared with the above studies, this study not only included more RCTs and a larger sample size, but also adopted a more appropriate multilevel meta-analysis model to analyze the multi-arm trials in the included studies to reduce the correlation caused by multi-arm trials, thereby improving the reliability and robustness of the study results and reducing the risk of bias.

While conducting the multilevel meta-analysis, to further clarify the effectiveness of different training methods in intervening inflammatory factors in the elderly, this study performed a corresponding network meta-analysis of four different intervention methods combined with the control group. The results of the SUCRA value ranking of intervention measures showed that, for IL-6, AT had the highest SUCRA value (84.6%), indicating a favorable ranking profile, with a 54.7% probability (PrBest) of being the best intervention. For TNF-α, AT also showed the highest SUCRA (97.5%) and a 90.6% probability of being best. For CRP, RT had the highest SUCRA (89.1%) with a 65.1% probability of being best. However, these rankings should be interpreted with caution given the very low GRADE evidence; for example, despite AT’s high SUCRA for IL-6, there remains a 45.3% chance that another modality (e.g., AT+RT or RT) is truly superior. Therefore, we refrain from labeling any modality as “the best” and instead present these as probabilistic estimates under the current evidence.

The results of pairwise comparisons showed that compared with the control group, AT had a significant anti-inflammatory effect on all three inflammatory factor outcome indicators; RT had a significant intervention effect on CRP, but its intervention effects on IL-6 and TNF-α did not reach statistical significance. In the comparisons between various exercise intervention methods, only the improvement effect of AT on TNF-α was significantly superior to that of MCEx, and the improvement effect of RT on CRP was significantly superior to that of MCEx, while the differences in effect sizes between the other groups did not reach statistical significance. The above results suggest that in clinical practice, when selecting exercise intervention methods for different inflammatory factors, AT can be preferred for improving IL-6 and TNF-α levels, and RT can be preferred for improving CRP levels; while MCEx has a relatively weak anti-inflammatory effect and can be used as an auxiliary intervention method. At the same time, it should be noted that except for specific combinations (AT vs MCEx for TNF-α, RT vs MCEx for CRP), the anti-inflammatory effects of other exercise intervention methods are not significantly different, and the clinic can flexibly select according to the actual situation such as the physical status and exercise tolerance of the elderly.

It is worth noting that the effect size results of the network meta-analysis are not completely consistent with those obtained by the multilevel meta-analysis, especially in terms of the intervention effect on IL-6: the multilevel subgroup analysis suggested that the intervention effect of AT+RT was superior to that of AT, while the result of the network meta-analysis model was the opposite. This difference may be caused by a variety of methodological factors: first, network meta-analysis can integrate direct and indirect comparison evidence within a unified model, thereby more comprehensively utilizing existing research information; while traditional subgroup analysis is only based on direct comparison and does not formally integrate indirect evidence, which may lead to bias in cross-intervention comparisons. Second, the network model included multi-arm interventions such as combined training, which formed new indirect comparison paths through common controls (such as the control group), which may have changed the effect estimates of aerobic and resistance training. In addition, the heterogeneity among studies in terms of exercise protocols, participant characteristics, and outcome measurement time may also have different impacts under different analysis methods. Nevertheless, neither the consistency test nor the node-splitting method found significant inconsistency in the network, suggesting that this difference is mainly due to the different methodological frameworks rather than data conflicts. In the future, more high-quality randomized controlled trials directly comparing aerobic and resistance training are needed to further clarify their relative efficacy.Therefore, we view the two sets of results as complementary. The MLMA provides robust direct-effect estimates that are easier to interpret for clinicians. The NMA offers a broader perspective by ranking all interventions simultaneously, albeit with greater uncertainty. For practical decision-making, the MLMA direct effects may be more relevant when choosing between an exercise modality and no exercise, while the NMA rankings are more useful when comparing active modalities head-to-head. Given the very low GRADE evidence, neither should be overinterpreted, and the convergence across methods (all active modalities are superior to control) is the most robust conclusion.

### 4.2 Subgroup analysis by health status

In addition, compared with the current existing studies, this study innovatively conducted a double subgroup analysis based on the different physical statuses of participants and different exercise intervention methods to explore the intervention effects of different exercise intervention methods on various inflammatory factors in the elderly with different physical statuses. The results showed that RT and AT+RT had statistically significant improvement effects on IL-6 and CRP in the elderly with chronic diseases, as well as on CRP in healthy people; while AT only had a statistically significant improvement effect on TNF-α in healthy people. This result can provide a referential evidence-based basis for the clinical formulation of individualized exercise anti-inflammatory intervention programs for the elderly with different physical statuses, and offer data support for subsequent relevant clinical practices and researches. It should be noted that due to the limited number of research literatures included in this study, it was not possible to further subdivide the elderly with chronic diseases (such as subdividing into different disease groups such as hypertension and sarcopenia). Therefore, clinically, when implementing exercise intervention for elderly patients with different specific diseases, the results of this study should be referred to with caution, and future relevant studies need to further subdivide different types of chronic diseases to provide more targeted evidence-based basis.

### 4.3 Potential biological mechanisms

Although the included RCTs did not directly measure molecular pathways, previous studies have proposed several mechanisms that may explain the anti-inflammatory effects of exercise. Regarding aerobic training, the nuclear factor κB (NF-κB) signaling pathway is one of the core molecular mechanisms regulating inflammatory responses. As a transcription factor, it binds to the inhibitory protein IκB and locates in the cytoplasm in a resting state. When cells are subjected to various stimuli (such as inflammatory factors, oxidative stress, etc.), IκB kinase (IKK) is activated, leading to the phosphorylation and degradation of IκB, and NF-κB is released and translocated into the nucleus, thereby initiating the transcription of pro-inflammatory genes (Zhang et al., 2021). Studies have shown that aerobic training can inhibit the activation of the NF-κB signaling pathway through multiple mechanisms. Among them, miR-146a, as an important negative regulator of inflammation, exerts anti-inflammatory effects by targeting key adapter molecules (such as IRAK1 and TRAF6) in the TLR and NF-κB signaling pathways, thereby reducing the nuclear translocation of NF-κB and the transcription of pro-inflammatory cytokines; aerobic training can up-regulate the expression of miR-146a and enhance its negative feedback regulatory effect on the NF-κB pathway (Yuan et al., 2025). Regarding resistance training, studies have shown that RT may reduce low-grade chronic inflammation by changing body composition and reducing the secretion of pro-inflammatory cytokines by adipose tissue (Lopes et al., 2020; Cheng and Yang, 2024); at the same time, resistance training may also reduce the secretion of pro-inflammatory cytokines by increasing the expression of anti-inflammatory cytokines. For example, some studies have found that resistance training may increase the level of IL-10, improve the IL-10/TNF-α ratio, and enhance the body’s anti-inflammatory capacity (Silva et al., 2018; Tomeleri et al., 2018a).These pathways are hypothesized based on external evidence; causal inference from the present data is not possible.

### 4.4 Clinical and research implications

In light of the very low GRADE evidence, high heterogeneity, and widespread risk of bias, the following suggestions are strictly preliminary. The data hint that aerobic training may be more likely to reduce IL-6 and TNF-α, and resistance training to reduce CRP, but ranking uncertainty is substantial (e.g., AT had only a 54.7% chance of being best for IL-6). For older adults with chronic diseases, RT or combined training could be considered for IL-6 and CRP, but these findings require replication. MCEx showed no consistent advantage. Definitive clinical recommendations cannot be made at this time; future high-quality trials are essential.

Statistically, exercise was associated with reductions in IL-6, CRP, and TNF-α, though the very low GRADE evidence limits confidence. In clinical terms, the observed SMD of 0.31 for IL-6 and 0.44 for CRP might translate into modest risk reductions (possibly in the range of 10–20%) for cardiovascular outcomes, but this interpretation is speculative given the indirectness of the biomarkers and the high heterogeneity across studies. For TNF-α, despite a statistically significant SMD of 0.40, the clinical relevance is unclear due to considerable heterogeneity and potential publication bias; therefore, the TNF-α findings should be viewed as exploratory.

## 5 Strengths and limitations

Potential methodological advancements of the present analysis. In addition to the larger sample size (82 RCTs, 6, 493 participants), this study offers several methodological features that may address limitations of previous meta-analyses, although the very low GRADE evidence warrants cautious interpretation. First, we employed a three-level multilevel meta-analysis to account for effect size dependency from multi-arm trials, which may reduce the risk of artificially inflated precision. Second, we conducted a network meta-analysis to integrate both direct and indirect evidence, allowing exploratory ranking of four exercise modalities and estimation of comparative effects that have rarely been compared head-to-head. Third, we performed a double subgroup analysis stratified by both exercise modality and health status, providing health-status-specific estimates that, while preliminary, may offer more granular insights. Fourth, we assessed rank uncertainty using probability plots rather than relying solely on SUCRA point estimates, which helps avoid overinterpretation of rankings. Finally, we conducted trim-and-fill sensitivity analyses and formal subgroup difference tests (with p-values reported) to better characterize heterogeneity. These approaches should be viewed as exploratory; the very low certainty of the underlying evidence means that all findings require replication in future high-quality trials.

Although this study systematically evaluated the effects of different exercise training methods on the levels of inflammatory markers in the elderly through rigorous systematic review, multilevel meta-analysis, and network meta-analysis, there are still several limitations that need to be considered when interpreting the study results:1) The original methodological quality of the included studies is a key factor affecting the strength of evidence in this meta-analysis. Although we strictly defined the inclusion criteria and only included randomized controlled trials (RCTs), the results of the bias risk assessment showed that most of the included studies (53 out of 82) had a high risk of bias. The main reason is that due to the particularity of exercise intervention, it is difficult to implement blinding for study participants and intervention implementers, which may introduce performance bias. At the same time, some studies did not clearly report the implementation of blinding for outcome assessors, resulting in potential detection bias. In addition, the information reported in some studies was insufficient, making the judgment of bias risk in terms of random sequence generation and allocation concealment “unclear”. These methodological deficiencies ultimately led to a very low GRADE evidence quality rating.2) The results of the multilevel meta-analysis for all outcome indicators (IL-6, CRP, TNF-α) in this study showed high statistical heterogeneity (all I² exceeded 75%). Although we explored the explanatory effect of factors such as exercise intervention type and participants’ health status on heterogeneity through subgroup analysis and double subgroup analysis, we still failed to fully explain the source of heterogeneity. Heterogeneity may originate from other insufficiently analyzed clinical characteristics, such as specific types of chronic diseases (e.g., sarcopenia, hypertension, diabetes), age stratification (e.g., elderly aged 60–70 years vs. those over 80 years old), details of intervention protocols (e.g., exercise intensity, duration, frequency), and differences in biomarker detection methods and unit conversion methods. Furthermore, the diversity of the control groups in the included trials—ranging from passive controls (e.g., ‘no exercise’ or ‘waiting list’) to active non-exercise controls (e.g., ‘nutritional intervention’ or ‘routine treatment’)—introduces significant clinical heterogeneity. While pooling these diverse modalities into a single ‘Control’ category was necessary to maintain network connectivity in the network meta-analysis, comparing an exercise intervention to an active control may yield a smaller effect size than comparing it to a passive control. This variation could potentially underestimate the true anti-inflammatory efficacy of the exercise interventions and heavily contribute to the observed statistical heterogeneity.3) The evidence network for the comparison of some intervention measures in the network meta-analysis is relatively sparse. For example, in the network structure of TNF-α, only 3 studies used MCEx as the exercise intervention method, resulting in wider confidence intervals for the estimated effect sizes of some comparisons (e.g., comparison between MCEx and other active interventions), and the results have a certain degree of uncertainty. At the same time, although no significant inconsistency was found by the overall inconsistency test and node-splitting method, due to the lack of direct evidence for some comparisons, the reliability of indirect comparisons still needs to be verified by more direct comparison results of multi-arm RCTs in the future.4) Furthermore, while we stratified participants into “healthy” and “chronic disease” categories, the “chronic disease” group encompassed heterogeneous conditions (e.g., hypertension, type 2 diabetes, COPD, sarcopenia) with potentially distinct inflammatory profiles and responsiveness to exercise. This broad categorization may have introduced residual confounding, as the magnitude of exercise-induced anti-inflammatory effects could vary across specific diseases. Future studies should conduct disease-specific subgroup analyses to better delineate the impact of different chronic inflammatory conditions.At the same time, due to the limited number of original studies, the time-dose effect of various exercises was not explored; if each exercise is divided into subgroups according to training duration and cycle to explore the time-dose effect, the number of studies that may be included in each subgroup will decrease, and the conclusion will be unreliable.

Based on the above limitations, to strengthen the evidence base and provide more precise guidance for clinical practice, future research can be carried out from the following key directions:1) Improve the methodological quality of original studies. Although it is difficult to implement blinding for participants and intervention implementers in exercise interventions, future randomized controlled trials should ensure that blinding is implemented for outcome assessors and statisticians to reduce detection bias. At the same time, researchers should detailedly report the protocols for random sequence generation and allocation concealment, as well as the reasons for loss to follow-up. Strictly following the Consolidated Standards of Reporting Trials (CONSORT statement) for reporting will help improve the transparency and reproducibility of studies; 2) Conduct more multi-arm randomized controlled trials to construct high-level direct evidence. Currently, the comparison of some intervention measures in the network meta-analysis (e.g., the efficacy comparison between aerobic training and resistance training on interleukin-6) mainly relies on indirect evidence. In the future, it is necessary to directly compare the efficacy of different exercise modes (e.g., aerobic training vs. resistance training vs. combined training) in the same study population to resolve the conclusion differences between different current analysis methods and improve the accuracy and credibility of treatment ranking; 3) In-depth exploration of the dose-response relationship of exercise anti-inflammatory effects. Future studies should systematically investigate how changes in parameters such as exercise intensity, total volume, and cycle affect the improvement of inflammatory markers, thereby providing a basis for formulating individualized and precise exercise prescriptions. In addition, using factorial design to explore the synergistic or superimposed effects of the combined application of exercise with nutritional supplements and drug interventions will be an important direction for exploring comprehensive anti-inflammatory strategies; 4) Emphasize long-term follow-up to evaluate the sustainability of intervention effects. Future studies should extend the intervention cycle and post-intervention follow-up time to clarify whether the exercise-induced anti-inflammatory effect can be maintained in the long term, and further explore its impact on hard endpoint indicators such as cardiovascular events, disability, and mortality; 5) Embed mechanism exploration in research design. It is encouraged to collect biological samples in randomized controlled trials to in-depth explore the molecular mechanisms underlying the anti-inflammatory effects of exercise. Focusing on the changes and interactions of key signaling molecules and cytokines such as nuclear factor κB, AMP-activated protein kinase, mammalian target of rapamycin, miR-146a, and interleukin-15 will help reveal the biological basis of the anti-inflammatory advantages of different exercise modes and provide a theoretical basis for the development of targeted exercise-mimetic drugs.

## 6 Conclusion

Based on very low GRADE certainty evidence, this systematic review and network meta-analysis suggests that exercise interventions may be associated with reductions in IL-6, CRP, and TNF-α levels in older adults. The available evidence, though very uncertain, hints at differential effects across exercise modalities: aerobic training showed the highest probability of being beneficial for IL-6 and TNF-α, whereas resistance training showed the highest probability for CRP. Health status may modify these effects; for older adults with chronic diseases, resistance or combined training appeared more promising for IL-6 and CRP, while aerobic training showed potential benefit for TNF-α in healthy older adults.

However, due to the very low certainty of the evidence (stemming from high risk of bias, substantial heterogeneity, and suspected publication bias), these findings should be considered exploratory and hypothesis-generating rather than definitive. No firm clinical recommendations can be made at this time. High-quality, adequately powered randomized controlled trials with blinded outcome assessment and standardized exercise protocols are urgently needed to confirm these preliminary observations and to establish optimal exercise prescriptions for inflammaging in the elderly.

## Data Availability

The original contributions presented in the study are included in the article/**Supplementary Material**. Further inquiries can be directed to the corresponding author.
