# Supplementary material for: Comparative efficacy of aerobic training, resistance training, combined training, and multicomponent exercise on interleukin-6, C-reactive protein, and tumor necrosis factor-α levels in older adults (≥60 years): a systematic review and network meta-analysis
Source: Front Physiol. 2026 Oct 14;17:1837579. doi: 10.3389/fphys.2026.1837579 (PMC13713320; doi:10.3389/fphys.2026.1837579)
Supplement: Supplementary file 1 [file SupplementaryFile1.docx]

IL-6

CRP

TNF-α

Supplementary Figure 1. Trim-and-fill funnel plots for the assessment of publication bias. (A) Interleukin-6 (IL-6); (B) C-reactive protein (CRP); (C) Tumor necrosis factor-α (TNF-α). White circles represent observed studies; filled (black) circles represent imputed studies added by the trim-and-fill procedure. The vertical dashed line indicates the adjusted pooled effect size (random-effects model). Pseudo 95% confidence limits are shown as dotted curves. The imputed number of studies and the adjusted effect sizes are reported below each plot.
