# Supplementary material for: Comparative efficacy of aerobic training, resistance training, combined training, and multicomponent exercise on interleukin-6, C-reactive protein, and tumor necrosis factor-α levels in older adults (≥60 years): a systematic review and network meta-analysis
Source: Front Physiol. 2026 Oct 14;17:1837579. doi: 10.3389/fphys.2026.1837579 (PMC13713320; doi:10.3389/fphys.2026.1837579)
Supplement: Supplementary file 2 [file Table1.docx]

Supplementary Table S1. Quantitative assessment of transitivity: comparison of key effect modifiers across direct comparison types.

| Direct comparison | Number of studies | Mean age (years) | % with chronic disease | Baseline IL-6 (pg/mL) | Baseline CRP (mg/L) | Baseline TNF-α (pg/mL) | Intervention duration (weeks) | % with high RoB |
| --- | --- | --- | --- | --- | --- | --- | --- | --- |
| AT vs. CON | 18 | 69.4 ± 5.2 | 38.9% | 2.8 ± 1.2 | 3.2 ± 1.5 | 4.1 ± 1.8 | 24.3 ± 16.7 | 61.1% |
| RT vs. CON | 28 | 70.1 ± 4.8 | 42.9% | 3.1 ± 1.5 | 3.5 ± 1.9 | 4.3 ± 2.1 | 18.9 ± 12.3 | 67.9% |
| AT+RT vs. CON | 12 | 69.8 ± 5.1 | 58.3% | 3.4 ± 1.8 | 3.8 ± 2.2 | 4.5 ± 2.4 | 26.5 ± 18.2 | 58.3% |
| MCEx vs. CON | 9 | 71.2 ± 4.5 | 44.4% | 2.9 ± 1.1 | 3.3 ± 1.6 | 4.0 ± 1.5 | 21.8 ± 14.5 | 55.6% |
| AT vs. RT | 5 | 68.5 ± 3.9 | 40.0% | 2.7 ± 0.9 | 3.0 ± 1.2 | 3.9 ± 1.3 | 20.4 ± 10.2 | 60.0% |
| AT vs. AT+RT | 4 | 67.9 ± 4.2 | 50.0% | 3.0 ± 1.0 | 3.4 ± 1.4 | 4.2 ± 1.6 | 22.5 ± 14.0 | 50.0% |
| AT+RT vs. RT | 3 | 69.2 ± 4.0 | 53.3% | 3.2 ± 1.2 | 3.6 ± 1.7 | 4.4 ± 1.9 | 24.0 ± 16.0 | 66.7% |
| **p-value** |  | **0.672** | **0.214** | **0.583** | **0.712** | **0.845** | **0.496** | **0.732** |

Note: Data are presented as mean ± SD or percentage. P-values from one-way ANOVA (continuous variables) or chi-square test (categorical variables). All p > 0.05, indicating no statistically significant differences across comparison types, supporting the transitivity assumption.*
