# Supplementary material for: Comparative efficacy of aerobic training, resistance training, combined training, and multicomponent exercise on interleukin-6, C-reactive protein, and tumor necrosis factor-α levels in older adults (≥60 years): a systematic review and network meta-analysis
Source: Front Physiol. 2026 Oct 14;17:1837579. doi: 10.3389/fphys.2026.1837579 (PMC13713320; doi:10.3389/fphys.2026.1837579)
Supplement: Supplementary file 3 [file Table2.docx]

Supplementary Table S2. Network balance diagnostics for IL-6, CRP, and TNF-α networks.

| Diagnostic metric | IL-6 | CRP | TNF-α |
| --- | --- | --- | --- |
| Number of interventions (nodes) | 5 | 5 | 5 |
| Number of possible pairwise comparisons | 10 | 10 | 10 |
| Number of comparisons informed by direct evidence | 7 | 8 | 6 |
| Network density (% of comparisons with direct evidence) | 70.0% (7/10) | 80.0% (8/10) | 60.0% (6/10) |
| Mean number of studies per direct comparison | 3.4 | 2.9 | 2.2 |
| Range of studies per comparison | 1–28 | 1–23 | 1–20 |
| Global inconsistency (design-by-treatment interaction p-value) | 0.345 | 0.689 | 0.849 |
| Sparsity index (1 - density) | 0.300 | 0.200 | 0.400 |

*Note: Sparsity index > 0.5 indicates a relatively sparse network. The TNF-α network is sparser than IL-6 and CRP, indicating that indirect comparisons for this outcome have greater uncertainty.*
